# DNA breathing and shape encode distinct mechanisms of transcription factor binding

**DOI:** 10.1093/bioadv/vbag287

**Published:** 2026-09-25

**Authors:** Tsu-Pei Chiu, Wei Yu Tang, Waqaas Butt, Boqiao Lai, Sheng Qian, Boian S Alexandrov, Jubao Duan, Xin He, Remo Rohs

**Affiliations:** Department of Quantitative and Computational Biology, University of Southern California, Los Angeles, CA 90089, United States; Department of Quantitative and Computational Biology, University of Southern California, Los Angeles, CA 90089, United States; Department of Human Genetics, University of Chicago, Chicago, IL 60637, United States; Toyota Technological Institute at Chicago, Chicago, IL 60637, United States; Department of Human Genetics, University of Chicago, Chicago, IL 60637, United States; SLIC.AI LLC, Santa Fe, NM 87507, United States; Center for Psychiatric Genetics, Endeavor Health Research Institute, Evanston, IL 60201, United States; Department of Psychiatry and Behavioral Neuroscience, University of Chicago, Chicago, IL 60637, United States; Department of Human Genetics, University of Chicago, Chicago, IL 60637, United States; Department of Quantitative and Computational Biology, University of Southern California, Los Angeles, CA 90089, United States; Department of Chemistry, University of Southern California, Los Angeles, CA 90089, United States; Department of Physics & Astronomy, University of Southern California, Los Angeles, CA 90089, United States; Thomas Lord Department of Computer Science, University of Southern California, Los Angeles, CA 90089, United States; Division of Medical Oncology, Department of Medicine, University of Southern California, Los Angeles, CA 90033, United States; Alfred E. Mann Department of Biomedical Engineering, University of Southern California, Los Angeles, CA 90089, United States

## Abstract

**Motivation:**

Interactions between transcription factors (TFs) and DNA play a key role in regulating gene expression. While these interactions are often viewed primarily as recognition of DNA sequence motifs, several studies have shown the limitation of this approach and highlighted the impact of physical properties of DNA. Prior studies have supported the importance of DNA shape features as determinant of TF–DNA recognition. Another physical property is DNA breathing, the spontaneous and transient opening of double-stranded DNA through thermal motions, which has not been systematically examined.

**Results:**

We analyzed *in vitro* TF–DNA binding data and found that DNA breathing features in motif regions are correlated with binding affinity, highlighting a role of locally and temporally melted DNA. Here, we compared contributions of DNA breathing and shape to TF binding. These analyses suggest that DNA breathing provides sequence-dependent information related to DNA structure not fully captured by DNA shape. Extending the analysis to ChIP-seq data, we found that breathing features are associated with TF binding *in vivo*, although the direction and magnitude of these associations vary across TFs. Our results support DNA breathing as an additional physical feature contributing to TF–DNA interactions alongside DNA sequence motifs and DNA shape.

**Availability and implementation:**

Data and analysis code are available at https://doi.org/10.17605/OSF.IO/UAHBJ and https://github.com/TsuPeiChiu/DNAbreathing.

## 1 Introduction

Spatial-temporal expression of genes is critical for development and cellular functions of organisms. Gene expression is regulated by the interactions of transcription factors (TFs) with their DNA target sites. A central problem in the field of gene regulation is to understand how these physical interactions are established. TF–DNA binding is often described through the recognition of sequence-specific DNA motifs. TF–DNA binding has been studied using both high-throughput experimental profiling and computational prediction (Inukai *et al*. 2017, Kim and Wysocka 2023). High-throughput *in vitro* binding assays, including protein-binding microarrays (PBMs) and high-throughput systematic evolution of ligands by exponential enrichment (HT-SELEX), profile TF binding preferences across large collections of synthetic or genomic DNA sequences, whereas *in vivo* approaches such as ChIP-seq identify TF-bound regions in cellular chromatin contexts (Inukai *et al*. 2017, Tognon *et al*. 2023). In parallel, computational methods have been developed to predict TF binding from DNA sequence, ranging from motif- and position weight matrix (PWM)-based models to machine-learning and deep-learning models that capture more complex sequence patterns across regulatory regions (Alipanahi *et al*. 2015, Zhou and Troyanskaya 2015, Kelley *et al*. 2016, Zhang *et al*. 2022, Tognon *et al*. 2023).

Despite the progress, important gaps remain in our understanding of how TFs interact with DNA. Genetic studies have shown that DNA variants affecting regulatory function or TF binding often reside outside of known motifs (Tehranchi *et al*. 2016, Gate *et al*. 2018). For example, one study found that although approximately 60% of putative causal variants for autoimmune diseases are located in enhancers, only 10%–20% directly alter TF motifs (Farh *et al*. 2015). Another study mapped genetic variants associated with TF binding and found that fewer than 0.9% of binding QTLs overlapped predicted motifs (Tehranchi *et al*. 2016). These observations suggest that features of DNA sequences beyond canonical motifs contribute to TF–DNA interactions.

To fill this gap, researchers have proposed that DNA variants may alter epigenomic features associated with regulatory DNA, thus affecting regulatory function (Klemm *et al*. 2019, Gaulton *et al*. 2023). For example, DNA variants can alter DNA methylation, which in turn can influence TF binding (Kribelbauer *et al*. 2020). Other mechanisms may involve local sequence composition, such as GC content near TF binding sites, or chromatin features such as nucleosome positioning (Segal *et al*. 2006, Struhl and Segal 2013, Inukai *et al*. 2017). Nevertheless, these mechanisms do not fully explain how sequence variation changes TF binding, and explicit evidence that DNA variants alter TF binding by directly changing these features remains limited. A complementary explanation is that intrinsic physical features of DNA contribute to TF binding. Indeed, we and others reported in previous studies that DNA shape features near motifs play an important role in determining TF–DNA interactions (Chiu *et al.* 2020). One study showed that incorporating DNA shape features within and near motifs improved the prediction of TF binding based on *in vitro* binding assays (Zhou *et al*. 2015). Other authors introduced the concept of DNA shape motifs, analogous to sequence motifs but defined by DNA shape features (Samee *et al*. 2019). Together, these studies indicate that DNA physical properties can contribute to TF binding beyond canonical sequence motifs, motivating the consideration of additional properties such as DNA breathing.

DNA shape is not the only factor in determining TF binding, in part because TFs rely on direct hydrogen-bond contacts with base pairs for motif recognition (Chiu *et al*. 2023). These hydrogen bonds are mainly formed in the major groove while DNA shape recognition is primarily described for electrostatic contacts in the minor groove (Rohs *et al*. 2009, Chiu *et al*. 2017). Other physical properties of the DNA double helix are probably also important. One such additional feature is DNA breathing (Guéron *et al*. 1987, Peyrard and Bishop 1989, Choi *et al*. 2004). DNA breathing refers to the spontaneous and transient opening of hydrogen bonds between complementary bases driven by thermal fluctuations. This transient opening can lead to local base-pair flipping and, when extended across multiple adjacent base pairs, DNA bubbles (Figure S1), representing a form of local DNA melting.

DNA breathing dynamics is generally sequence-specific, with the probability of DNA bubble formation and other breathing features influenced by local sequence context. This sequence dependence reflects local duplex stability, which is shaped by both base-pair hydrogen bonding and nearest-neighbor stacking interactions. A/T base pairs form two hydrogen bonds, whereas G/C base pairs form three hydrogen bonds. However, base-pair composition alone does not fully determine duplex stability, because stacking interactions between adjacent base pairs also provide major contributions to DNA thermal stability. Together, base-pair identity and local stacking context influence the energetic cost of transient base-pair opening and bubble formation (SantaLucia 1998, Choi *et al*. 2004, Yakovchuk *et al*. 2006). Earlier studies showed that DNA breathing dynamics can influence TF binding (Alexandrov *et al*. 2012, Nowak-Lovato *et al*. 2013). Importantly, sequence variants influencing DNA breathing may affect TF binding and regulatory functions of DNA. These earlier studies, however, were limited to a small number of TFs or DNA variants. Specifically, a mechanistic understanding requires a rigorous statistical analysis of the contribution of DNA breathing to TF binding. Furthermore, such analysis requires a quantitative comparison to models based on nucleotide sequence and DNA shape.

A recent study showed that incorporating DNA breathing features in sequence-only models provides a modest improvement in predicting TF binding targets (Kabir *et al*. 2024). However, this study has several limitations that prompted a reassessment of the claim that DNA breathing independently affects TF–DNA binding. Given the complex and non-linear nature of deep neural networks, the resulting models from the study are difficult to interpret. This earlier study was unable to identify DNA breathing as a cause for the reported model performance and failed in uncovering the mechanisms of how DNA breathing can contribute to protein–DNA binding (Kabir *et al*. 2024).

Another recent study of the impact of DNA flexibility on TF binding uses an alternative approach to define conformational flexibility based on a set of features that do not include DNA breathing (Dey *et al*. 2026). Molecular dynamics (MD) simulations have also been used to probe DNA conformational flexibility (Battistini *et al*. 2026). However, despite extensive computational resources devoted to deriving physical properties from these simulations, a recent study reports fraying or melting at the terminal ends of DNA oligonucleotides (Battistini *et al*. 2026). These finite DNA fragments, only 20 bp in length, do not accurately represent the nature of genomic DNA. Although the study reports these fraying or melting observations as rare events, they are more likely simulation artifacts that fail to capture biologically relevant features of DNA breathing, as discussed here.

Our goal in this study was to perform a comprehensive statistical analysis integrating established DNA features, such as breathing, shape, TF motifs, and TF-binding data, to examine the role of DNA breathing in TF–DNA interactions. Our main hypothesis is that DNA breathing features at motif sites or within motif-containing target regions may influence the strength of TF–DNA interactions. For example, as a mechanistic hypothesis, a DNA bubble may facilitate TF binding by opening adjacent DNA base pairs transiently. In such a scenario, although TFs typically bind double-stranded DNA through contacts in the major and minor grooves, the presence of a bubble opens base pairs within the core motif and allows additional or alternate interactions with the base-pairing edge of the nucleotides, potentially including amino acid–base hydrogen bonding with donors and acceptors otherwise involved in base pairing. Such interactions might increase binding affinity depending on which side chains engage in interactions with DNA, and where they are located on the protein surface.

We used two categories of DNA breathing features derived from DNA breathing dynamics in physical simulations based on the Extended Peyrard-Bishop-Dauxois (EPBD) approach (Kabir *et al*. 2023). The first feature (‘flipping probability’) captures the local base-pair-level breathing profiles, while the second feature (‘bubble probability’) captures the breathing profile over an extended sequence window. Specifically, flipping probability denotes the probability that a base may be transiently flipped out of the double helix. At the extended sequence level, DNA breathing may create a bubble that spans multiple adjacent base-pair positions. Bubble probability captures the statistical likelihood of bubble formation at different locations within a given nucleotide sequence, thereby describing both bubble location and length. We note that bubble formation probability depends on the length and width threshold used to define bubbles (see Methods). Both features capture the tendency of opening intra-base pair hydrogen bonds and weakening of nearest-neighbor stacking interactions that can lead to local melting of the double helix.

In this study, we used three complementary datasets to investigate how DNA breathing may contribute to TF–DNA interactions. First, we used genomic-context PBM (gcPBM) data for basic helix-loop-helix (bHLH) TFs, which measure *in vitro* TF binding to DNA fragments that preserve native genomic flanking contexts (Gordân *et al*. 2013). Second, we analyzed HT-SELEX data to evaluate breathing–binding associations across a broader set of TFs under controlled *in vitro* selection (Yang *et al*. 2017). Together, these *in vitro* datasets allowed us to assess TF–DNA binding directly while reducing the influence of cellular factors such as chromatin accessibility, chromatin state, and TF abundance (Xin and Rohs 2018, Klemm *et al*. 2019, Kribelbauer *et al*. 2019). Finally, we extended the analysis to ChIP-seq data to evaluate whether similar associations can be observed *in vivo* across diverse TF families.

Because DNA breathing is sequence-dependent and may be related to other DNA physical properties, we further compared breathing features with DNA shape features. DNA shape has been extensively studied as an additional layer of TF–DNA recognition beyond nucleotide sequence, with parameters such as minor groove width (MGW), propeller twist (ProT), Roll, and helix twist (HelT) capturing local DNA geometry that can contribute to TF binding specificity (Zhou *et al*. 2013, 2015, Li *et al*. 2024). However, DNA shape features primarily describe the preferred structural geometry of the double helix, whereas DNA breathing describes the propensity of base pairs to transiently open. This comparison allowed us to ask whether DNA breathing provides information that is distinct from, or complementary to, established shape-based descriptions of TF–DNA recognition.

Overall, our analyses show that DNA breathing features at motif sites or within motif-containing target regions are associated with TF binding across multiple experimental settings. These associations are not uniform across TFs, suggesting that different TFs may use or tolerate locally opened DNA states in different ways. Together, our results support DNA breathing as an additional physical feature contributing to TF–DNA interactions, complementary to DNA sequence motifs and DNA shape.

## 2 Methods

### 2.1 *In vitro* TF binding data from gcPBM and HT-SELEX

We investigated TF binding affinities using two datasets. The first dataset, derived with gcPBM binding assays, included 23,028 sequences, each 36-base pairs (bp) in length, with relative binding affinity on a scale from 0 to 1 for the bHLH TF complexes: Max-Max homodimer (MAX), Mad2-Max heterodimer (MAD), and c-Myc-Max heterodimer (MYC) (Gordân *et al*. 2013). The second dataset was derived from HT-SELEX experiments, where oligonucleotides of 14, 20, 30, or 40 bp in length, excluding sequence-invariant terminal ends, were used to measure TF–DNA binding. After quality-control filtering, this dataset included 512 datasets covering 215 human and mouse TFs from 27 TF families, with binding represented by enrichment-based *M*-word scores (Yang *et al*. 2017).

### 2.2 *In vivo* TF binding data from ChIP-seq

The third dataset comprised 886 625 sequences, each 200-bp long, annotated with ChIP-seq peak data from the DeepSEA study (Zhou and Troyanskaya 2015) for 64 different TFs. Here, binding affinity was dichotomous, recorded as either 1 (peak present, bound) or 0 (peak absent, unbound). The positive and negative labels were taken from the DeepSEA-derived binary annotation matrix. For each TF, sequences with a ChIP-seq peak label for that TF were treated as positive examples, whereas sequences from the same 200-bp sequence collection without a peak label for that TF were treated as negative examples for that TF. Thus, a sequence could be positive for one TF and negative for another. The sequence collection was not composed of random genomic background sequences; instead, it included sequences bound by at least one TF in the original datasets. However, negative examples were not explicitly matched to positive examples by distance to transcription start sites or GC content. Positive and negative label counts for each TF are summarized in Table S2, available at supplementary material  *Bioinformatics Advances* online. The subset used for the ChIP-seq bubble-probability analysis is summarized in Table S3, available at supplementary material  *Bioinformatics Advances* online.

We retained the standardized 200-bp sequence windows from the DeepSEA dataset because the bound/unbound labels were defined for these sequence examples. This window size captures TF motifs together with nearby flanking context, while avoiding longer distal sequence context that could dilute motif-proximal DNA breathing signals. In the ChIP-seq analyses, breathing features were summarized locally at motif-matched positions for flipping probability, or near motif matches for bubble probability, rather than averaged across the full 200-bp window.

### 2.3 Computational motif scanning

We utilized FIMO from the MEME Suite (Grant *et al*. 2011) to perform the motif scans, using PWMs obtained from the JASPAR2022 database (Castro-Mondragon *et al*. 2022). We categorized motif matches into two strength levels: strong (*P*-value < 1 × 10^−^^4^) and weak (*P*-value between 1 × 10^−^³ and 1 × 10^−^^4^). Unless otherwise stated, a “motif match” refers to any FIMO hit with *P*-value < 1 × 10^−^³, including both strong and weak motif matches. This process allowed us to pinpoint exact bp positions containing a motif for a given TF, and a corresponding *P*-value for that motif match. Since JASPAR does not have a motif PWM for MAD, the gcPBM data for MAD were excluded from motif-based flipping analyses, which required FIMO motif scanning. However, MAD was included in the bubble-presence comparison because this analysis did not require motif information. Similarly, 20 of the 64 TFs in our ChIP-seq dataset did not have motif PWMs in JASPAR and were excluded from analysis. The exact versioned JASPAR matrix identifiers used in the gcPBM and ChIP-seq FIMO analyses are provided in Table S4, available at supplementary material  *Bioinformatics Advances* online and archived in the Open Science Framework (OSF) repository associated with this study (see data availability statement).

### 2.4 Defining DNA breathing features

Our breathing data was generated using pyDNA-EPBD, a Python-based MCMC simulation package that uses the EPBD nonlinear DNA model to describe DNA dynamic features (Kabir *et al*. 2023). The simulation parameters were set to the standard settings recommended by the package, with the temperature set to 310 K, preheating equilibration steps set to 50 000, and simulation production steps set to 80 000. The package outputs a three-dimensional tensor of DNA bubble probabilities, where each value in the tensor is defined as the probability of a certain bubble of length *l* (measured horizontally across the DNA molecule in bp length) with displacement *thr* (measured in Å) forming at a base at position *n*. Here displacement means the distance of a bp position in an open bubble from what its position would be if the bubble were not present. The flipping probability feature is defined as probability that a specific base is flipped out of the double helix, and a given base is considered flipped based on a certain displacement threshold in Å. We calculated flipping probability at a displacement threshold of 0.5 * √2 Å. This threshold follows the recommended pyDNA-EPBD setting and was kept fixed across datasets to ensure consistent comparisons. Due to the sheer volume of ChIP-seq sequences used to compute the flipping probability correlations in our analysis, we used pre-generated features. For the gcPBM sequences, we computed flipping probability ourselves since it was computationally feasible. The simulation also generates coordinate distance values (as well as the square of those values) for each bp in the input sequence, which we used as alternative features to define flipping.

To define DNA bubbles, we used a threshold based on prior EPBD-based DNA breathing work (Alexandrov *et al*. 2012). Specifically, a bubble was defined as a region with displacement >3.5 Å and bubble length >10 bp. The displacement threshold identifies substantially opened bp states, whereas the length threshold focuses the analysis on extended local opening events rather than isolated bp fluctuations. Using these criteria, we sliced the pyDNA-EPBD tensor to obtain one bubble-probability value per bp position. We then used the 95th percentile of bubble-probability values as a conservative relative threshold to categorize whether a bubble was present at a given position or sequence. This criterion was applied consistently across bound and unbound groups and resulted in ∼20% bubble-positive sequences in the gcPBM data and ∼40% in the ChIP-seq data.

Computing DNA bubble features is resource-intensive but highly parallelizable because each sequence can be simulated independently. For the ChIP-seq bubble-probability subset, generating bubble features for approximately 38 000 sequences 200 bp in length required about 36 hours of wall-clock time on the University of Chicago Midway3 computing cluster using 100 parallel jobs. Each job used 24 CPU cores with 2 GB memory per core on AMD EPYC 7702 processors. Runtime scales with the number and length of sequences and the number of simulation steps. Thus, genome-wide applications are feasible for targeted motif- or peak-centered regions on high-performance computing systems, but full genome-wide bubble simulations remain computationally demanding and would benefit from additional filtering or parallelization.

### 2.5 Testing correlation of flipping features with *in-vitro* binding affinity

For the first part of the correlation analysis, we averaged flipping probability across all motif positions in a sequence, resulting in a single average flipping probability per sequence that we then correlated against the binding affinity of the sequence. For the second part of the analysis, we took the flipping probability at each individual bp location in a motif and correlated these values against binding affinity of the sequences to yield correlation results at each bp position of the motif.

### 2.6 Testing correlations between flipping and DNA shape features

To examine the relationship between DNA breathing and shape, we compared mean flipping and bubble probabilities with four standard DNA shape features: MGW, ProT, Roll, and HelT (Zhou *et al*. 2013). Following the pentamer-based sequence representation used in DNAshapeR (Chiu *et al*. 2016), we enumerated 512 nonredundant double-stranded pentamers by treating reverse-complement sequences as equivalent. For each pentamer, we calculated mean flipping probability and mean bubble probability and obtained corresponding DNA shape parameters from sequence-based shape predictions. Pearson and Spearman’s rank correlation coefficients were computed between each breathing feature and each shape parameter across pentamers. To visualize sequence-context effects, pentamers were annotated by dinucleotide-step class, with pyrimidine–purine (YpR) subclasses including CpG, TpA, and CpA/TpG highlighted separately.

### 2.7 Testing correlation of bubble presence with *in-vitro* binding affinity

We compared gcPBM-derived binding affinity of sequences with detectable bubbles, denoted bubble-positive sequences, and sequences without detectable bubbles, denoted bubble-negative sequences. For each TF complex, we conducted a Wilcoxon rank sum test to determine whether the binding affinity significantly differed between the two groups.

### 2.8 Training multiple linear regression models

We used a TF binding dataset obtained from HT-SELEX experiments, which contains binding measurements for 215 TFs (Yang *et al*. 2017). From this dataset, we randomly sampled 38 TFs across 27 TF families, comprising 241 210 DNA sequences across the selected TFs. For each DNA sequence, we generate three feature sets:

- Sequence features (1-mer): each position is one-hot encoded, yielding *l* × 4 features per sequence, where *l* denotes the sequence length. The HT-SELEX sequence windows were preprocessed in the original dataset by identifying a core TF-binding motif and centering each window on that motif; sequences containing multiple core-motif instances were excluded. Thus, nucleotide positions are aligned relative to the most probable binding site. The 1-mer model is therefore a position-specific additive sequence model rather than a model based only on global nucleotide composition.
- DNA shape features: we run Deep DNAshape (Li *et al*. 2024) to compute shape parameters, producing *l* × 7 intra-bp features and (*l*–1) × 6 inter-bp features. The seven intra-bp features are MGW, Shear, Stretch, Stagger, Buckle, ProT, and Opening, and the six inter-bp features are Shift, Slide, Rise, Tilt, Roll, and HelT.
- DNA breathing features: we run pyDNA-EPBD (Kabir *et al*. 2023) to obtain the bubble and flipping profiles. For both profiles, we used the per-base probability as described in the section ‘Defining DNA breathing features’. This yields *l* × 2 features per sequence.

DNA shape and breathing features are standardized to zero mean and unit variance, performed separately for each TF. For each feature *c* and position *i*, values are normalized as


$$\hat{x_{c,i}}=\frac{x_{c,i}-\mu_{c,i}}{\sigma_{c,i}}$$

where $\mu_{c,i}$ and $\sigma_{c,i}$ denote the mean and standard deviation of feature c at position i.

For each TF, we then trained several multiple linear regression (MLR) models with L2 regularization to predict binding scores from different sets of features. Model performance is assessed using 10-fold cross-validation, and the regularization hyperparameter is selected via grid search on the full dataset. Performance is reported as the mean coefficient of determination (*R^2^*) averaged across the 10 cross-validation folds.

### 2.9 Testing correlation of flipping features with *in-vivo* binding affinity

We replicated the two methodologies used for the gcPBM data using 44 different TFs, with some TFs having more than one motif (PWM) with different consensus sequences.

### 2.10 Testing correlation of bubbles with *in-vivo* binding affinity

For each sequence, we first isolated the strongest motif match (by *P*-value) in that sequence and then assessed if there is a nearby bubble at different thresholds (5, 10, or 20 bp). We then performed logistic regressions to test the effect of bubble presence near a motif (binary variable) of a sequence on its binding affinity.

## 3 Results

### 3.1 DNA flipping probability feature correlates with *in-vitro* TF binding

We first examined the relationship between DNA breathing and TF binding using the flipping probability feature, which is defined in the Methods subsection “Defining DNA breathing features.” We hypothesized that flipping probability profiles at the motif locations are associated with TF binding. To test this, we used gcPBM data of two bHLH TF complexes: MAX and MYC (Gordân *et al*. 2013) (Table S1, available at supplementary material  *Bioinformatics Advances* online). For each TF complex, we considered all sequences that contain at least one match to the TF motif, as defined in the Methods subsection “Computational motif scanning.” For any such sequence, we computed the average flipping probability across all nucleotide positions in the motif match. When a sequence contained multiple motif matches, all the matched positions were included for computing flipping probability. We observed statistically significant positive correlations between average flipping probability and DNA binding affinity for both MAX and MYC, with Pearson correlations of *r *= 0.26 for MAX and *r *= 0.33 for MYC (*P *= 1.14 × 10^−29^ and *P *= 1.77 × 10^−68^), respectively (Fig. 1A). The results suggest that sequences with higher average flipping probability at motif sites tended to exhibit higher binding affinity.

**Figure 1:**
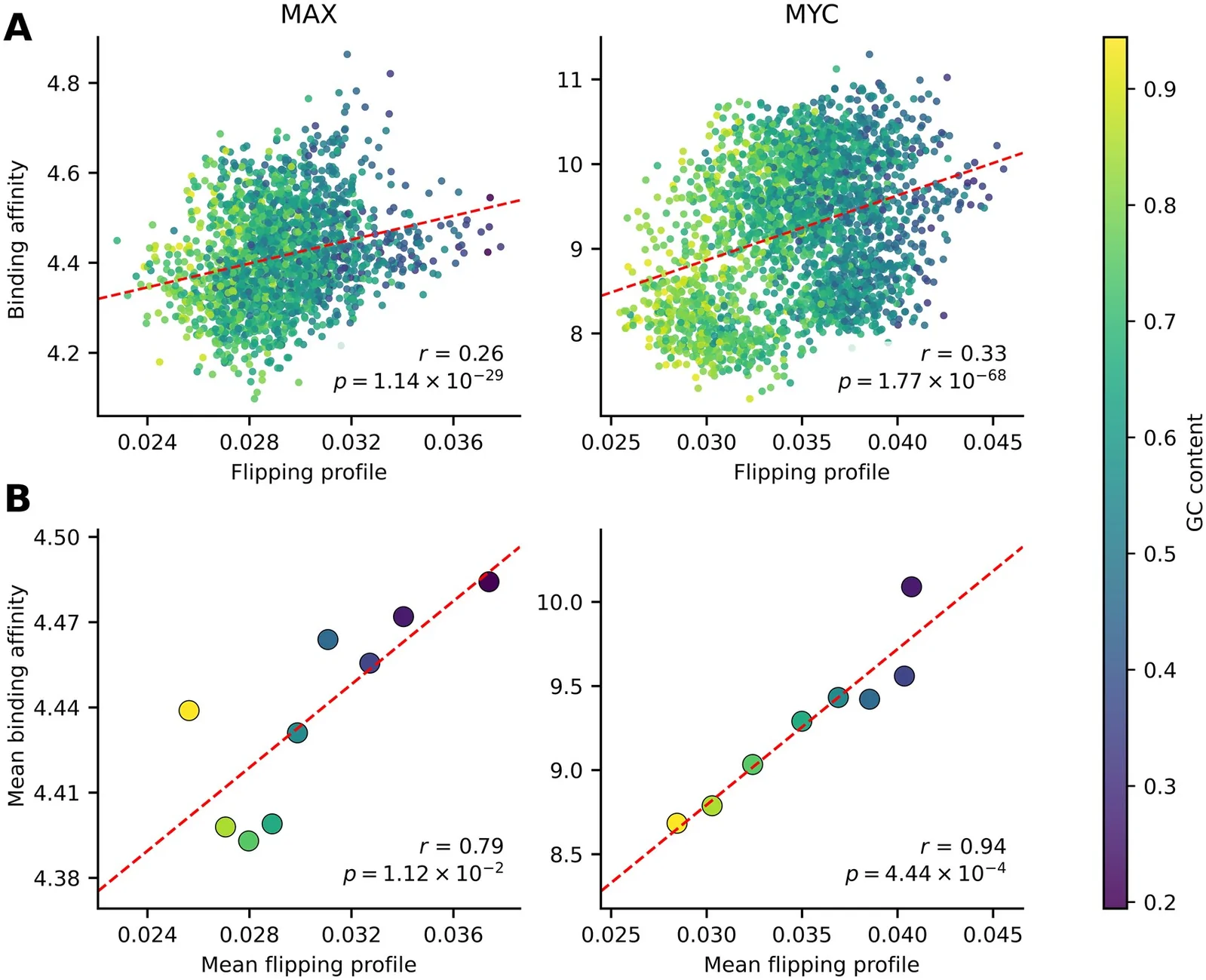
DNA flipping probability at motif sites correlates with *in-vitro* TF binding affinity. (A) Binding affinity versus average flipping probability across motif-matched positions for MAX (left panel) and MYC (right panel). Each point represents one motif-containing sequence, colored by GC content; red dashed lines indicate linear fits. (B) GC-content-binned analysis for MAX (left panel) and MYC (right panel) showing the relationship between mean flipping probability and mean binding affinity.

Because GC content influences base-pairing stability through hydrogen bonding, and YpR dinucleotide steps can alter local base stacking geometry and flexibility, we further examined whether these sequence-composition features were related to the observed flipping–binding association. Grouping sequences by GC content strengthened the positive association between the flipping profile and binding affinity, with *r* increasing from 0.26 to 0.79 for MAX and from 0.33 to 0.94 for MYC (Fig. 1B).

A complementary analysis using YpR-count bins showed a similar increase in correlation, with *r *= 0.85 for MAX and *r *= 0.87 for MYC (Fig. 2). These binned analyses suggest that the flipping–binding relationship reflects local base-pairing stability, modulated by GC-dependent hydrogen bonding and YpR-dependent stacking interactions.

**Figure 2:**
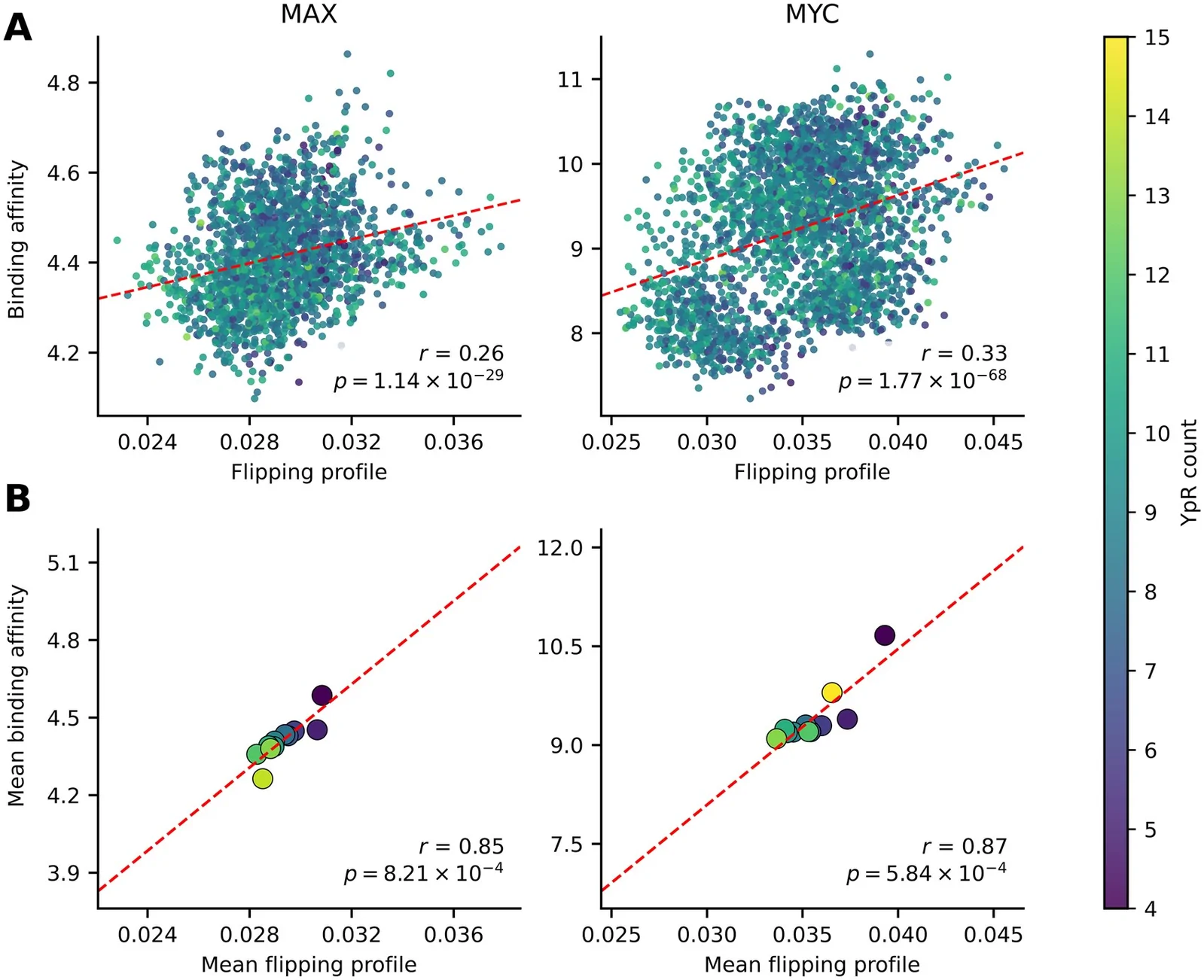
YpR-count analysis of the relationship between DNA flipping probability and *in-vitro* TF binding affinity. (A) Binding affinity plotted against the average flipping probability across motif-matched positions for MAX (left panel) and MYC (right panel). Each point represents one motif-containing sequence, colored by YpR count; red dashed lines indicate linear fits. (B) YpR-count-binned analysis showing mean binding affinity versus mean flipping probability for MAX (left panel) and MYC (right panel). Pearson correlation coefficients and *P*-values are shown in each panel.

### 3.2 DNA bubble probability is associated with *in-vitro* TF binding affinity

We next examined whether the presence of DNA bubble features within target regions is associated with binding affinity for MAX, MYC, and MAD. This initial bubble-presence comparison did not require motif information, so MAD could be included despite the lack of a corresponding PWM in the JASPAR database. Across all three TF complexes, sequences with detectable bubbles (denoted as bubble positive sequences) generally exhibited higher binding affinity than those without detectable bubbles (denoted as bubble negative) (Figure S2, available at supplementary material  *Bioinformatics Advances* online). The difference in the distributions of binding affinities was statistically significant for all three TF complexes, based on Wilcoxon rank sum test. These results suggest that the presence of DNA bubbles is associated with higher TF binding affinity, consistent with the hypothesis that bubbles may contribute to binding.

### 3.3 Relationship between DNA breathing features and DNA shape parameters

To examine whether DNA breathing features reflect DNA shape, we first compared the mean flipping and bubble probabilities with MGW across pentamer sequence contexts. MGW is a widely used DNA shape feature that captures local groove geometry and can influence protein–DNA recognition by affecting electrostatic potential readout (Rohs *et al*. 2009, Chiu *et al*. 2017). However, both breathing metrics showed only weak overall correlations with MGW, with Pearson and Spearman’s rank correlations of *r* = −0.090 and *ρ* = −0.059 for flipping probability, respectively, and *r* = −0.081 and *ρ* = −0.022 for bubble probability (Fig. 3A). These weak correlations indicate that the sequence-dependent variation in DNA breathing is not simply explained by MGW alone. When dinucleotide-step classes were highlighted, YpR subclasses showed distinct distributions in the breathing–MGW space: CpG-containing contexts tended to show lower breathing values, TpA-containing contexts tended to show higher breathing values, and CpA/TpG-containing contexts occupied an intermediate range. In contrast, breathing values and MGW of purine–purine (RpR)- and pyrimidine–pyrimidine (YpY)-containing contexts were more broadly distributed (Fig. S3, available at supplementary material  *Bioinformatics Advances* online). These observations suggest that DNA breathing is influenced by local sequence context and nearest-neighbor interactions, rather than being reducible to MGW as a single static shape descriptor. The lower breathing propensity of CpG-containing contexts is consistent with the greater stability of G/C bp, whereas the higher breathing propensity of TpA-containing contexts is consistent with relatively weak stacking at TpA bp steps.

**Figure 3:**
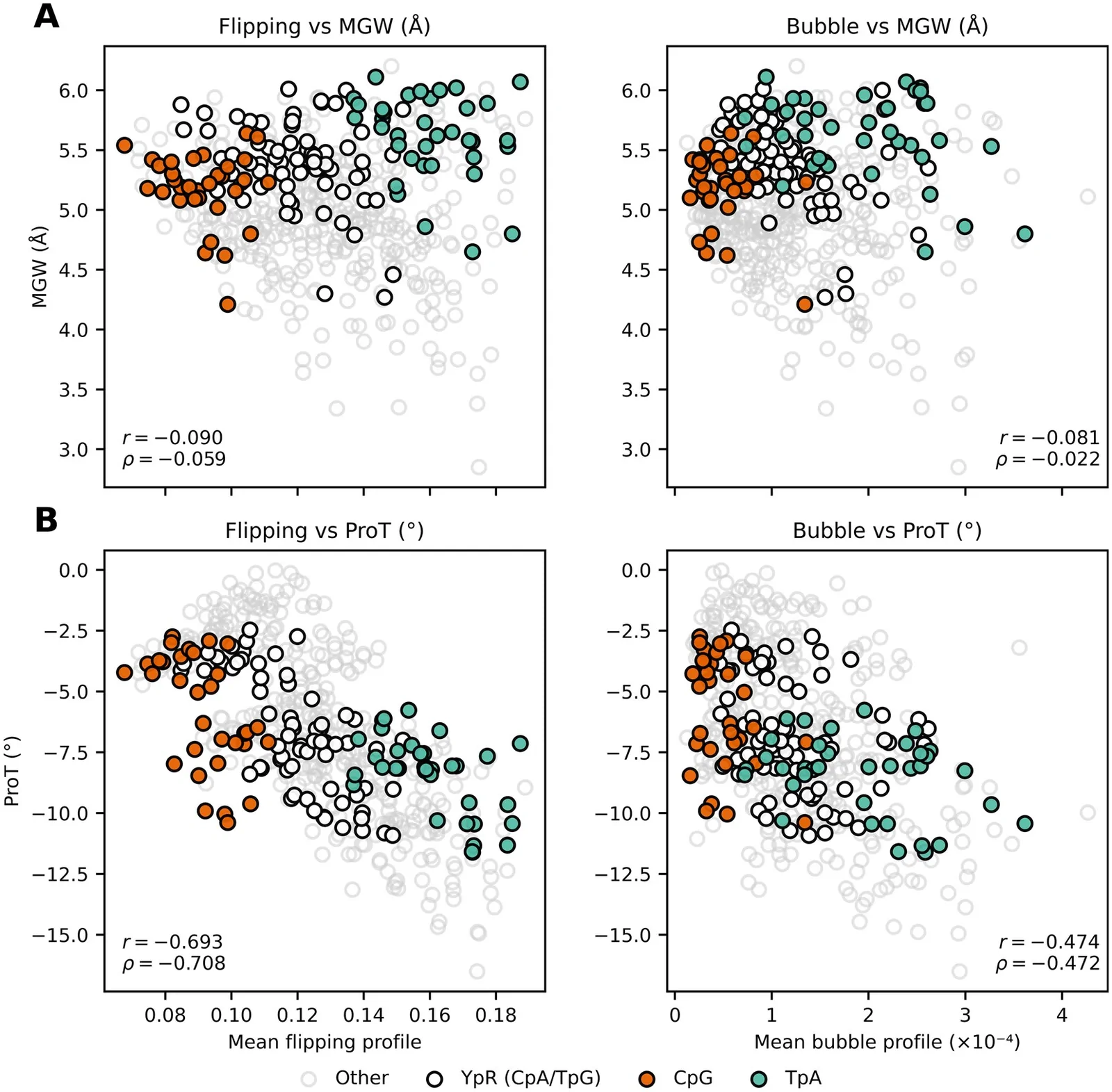
Relationship between DNA breathing features and DNA shape parameters across pentamer sequence contexts. (A) Mean flipping probability (left panel) and mean bubble propensity (right panel) plotted against minor groove width (MGW) across 512 pentamer contexts. (B) Mean flipping probability (left panel) and mean bubble probability (right panel) plotted against propeller twist (ProT). CpA/TpG, CpG, and TpA-containing contexts are highlighted, while all other contexts are shown in gray. Pearson and Spearman’s rank correlation coefficients are shown in each panel. MGW showed weak overall correlations with both breathing features, whereas ProT showed stronger negative associations, especially with flipping probability.

We next examined ProT, an intra-bp shape parameter that reflects local bp geometry. In contrast to MGW, ProT showed a clear negative association with DNA breathing features. Pentamer contexts with higher mean flipping probability tended to have more negative ProT values, with Pearson and Spearman’s rank correlations of *r* = −0.693 and *ρ* = −0.708, respectively (Fig. 3B). Bubble probability also showed a negative association with ProT, although the relationship was weaker, with Pearson and Spearman’s rank correlations of *r* = −0.474 and *ρ* = −0.472, respectively (Fig. 3B). This difference is informative because flipping represents a more local base-exposure or unstacking event, whereas bubble formation requires the cooperative opening of multiple adjacent bp. Therefore, ProT appears to be more directly coupled to flipping than to bubble formation.

The distribution of dinucleotide-step classes further supported this interpretation. CpG-containing contexts were enriched at lower flipping and bubble values, TpA-containing contexts were enriched at higher breathing values and more negative ProT, and CpA/TpG-containing contexts occupied an intermediate range. These patterns suggest that the relationship between ProT and DNA breathing is shaped by nearest-neighbor sequence context, likely through local bp geometry and stacking interactions. Additional analyses highlighting RpR- and YpY-containing contexts showed broad distributions across the ProT–breathing space, indicating that the negative ProT–breathing relationship is not restricted to YpR steps alone (Figure S4, available at supplementary material  *Bioinformatics Advances* online). However, the YpR subclasses provide clearer sequence-specific landmarks, with CpG, CpA/TpG, and TpA contexts occupying distinct regions of ProT-affected DNA breathing.

To further examine whether this pattern extends to other DNA shape parameters, we also compared the mean flipping and bubble probabilities with HelT and Roll across the same pentamer sequence contexts (Figure S5, available at supplementary material  *Bioinformatics Advances* online). HelT showed weak positive overall correlations with DNA breathing, with Pearson and Spearman’s rank correlations of *r *= 0.182 and *ρ* = 0.143 for flipping probability and *r *= 0.187 and *ρ* = 0.161 for bubble probability, respectively. Roll showed weak negative correlations with flipping probability and little overall correlation with bubble probability, with Pearson and Spearman’s rank correlations of *r* = −0.175 and *ρ* = −0.172 and *r* = −0.039 and *ρ* = −0.023, respectively. These results indicate that neither HelT nor Roll alone explains the sequence-dependent variation in DNA breathing.

Although the overall correlations were weak, Roll revealed clear sequence-class patterns. YpR-containing pentamers, including CpG, CpA/TpG, and TpA contexts, were enriched in the positive Roll region, whereas YpY-containing pentamers tended to occupy the negative Roll region (Figure S6, available at supplementary material  *Bioinformatics Advances* online). This suggests that Roll captures broad local bp step geometry. Importantly, TpA- and CpG-containing contexts were not clearly separated by Roll, even though they showed distinct breathing propensities. CpG-containing contexts tended to show lower breathing values, consistent with greater duplex stability, whereas TpA-containing contexts tended to show higher breathing values, consistent with weaker stacking and greater local flexibility. Thus, Roll captures a shared YpR-like closed-state geometry, while DNA breathing further captures differences in local opening propensity.

Together, these results suggest that DNA breathing is related to DNA shape but is not reducible to any single static shape parameter. Instead, breathing reflects dynamic, sequence-dependent opening propensity shaped by local bp stability and nearest-neighbor interactions.

### 3.4 Comparing DNA breathing and shape in TF-binding affinity predictions

To further test whether DNA breathing features provide predictive information beyond position-specific mononucleotide sequence features, we compared regression models using different feature sets on HT-SELEX data. This analysis included 38 randomly selected TFs from 27 TF families, comprising 241 210 DNA sequences in total. We first used a baseline model containing only mononucleotide sequence features, referred to here as the 1-mer model. Adding DNA breathing features to this sequence baseline improved the prediction of relative binding affinity for most TFs, with the *R^2^* value increasing in 33 of 38 TFs. The mean *R^2^* increased from 0.503 for the 1-mer model to 0.527 for the 1-mer plus breathing model, and the paired improvement was statistically significant by Wilcoxon signed-rank test (*P *= 0.0002; Fig. 4A). Although the average improvement was modest, the consistent direction of improvement across TFs from diverse classes suggests that DNA breathing captures sequence-dependent information that is not fully represented by position-specific mononucleotide sequence features alone.

**Figure 4:**
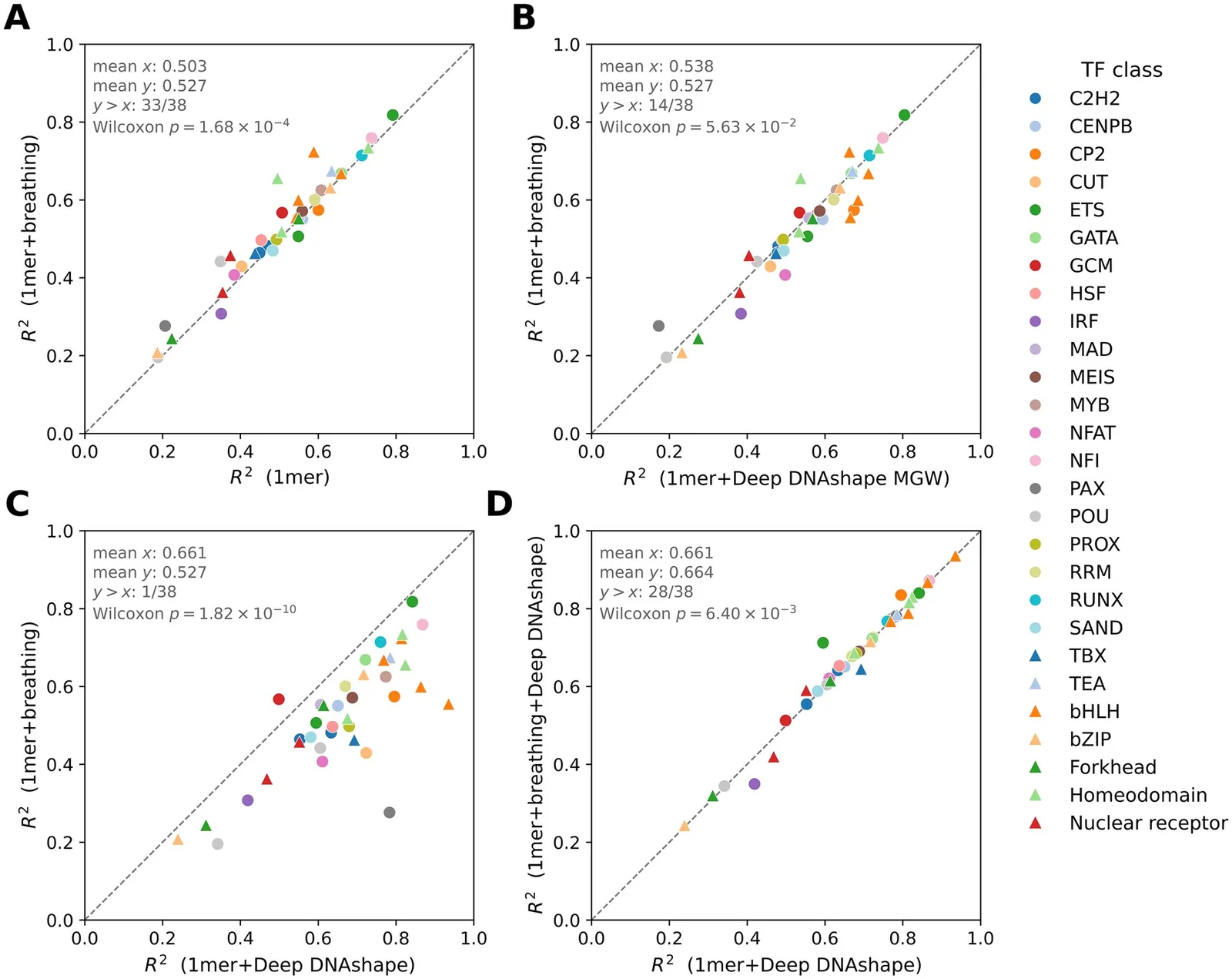
Comparison of 10-fold cross-validated *R*² values for 38 TFs sampled across 27 TF classes from an HT-SELEX dataset of 215 TFs. For each TF, an MLR model with L2 regularization was trained to predict HT-SELEX-derived binding scores. Each point represents one TF, colored and shaped by TF class. (A) 1-mer + breathing model versus 1-mer model. (B) 1-mer + breathing model versus 1-mer + MGW model. MGW was predicted by Deep DNAshape. (C) 1-mer + breathing model versus 1-mer + 13 DNA structural features predicted by Deep DNAshape. (D) 1-mer + breathing + 13 DNA structural features model versus 1-mer + 13 DNA structural features model. The dashed line indicates equal performance. Plot statistics report the mean *R*² for each TF according to the model denoted for the respective axis, the number of TFs for which the *y*-axis model outperformed the *x*-axis model, and the *p*-value from a two-sided Wilcoxon signed-rank test on paired per-TF *R*² values.

We next compared the model combining 1-mer sequence features with breathing features to a model combining 1-mer sequence features with the MGW feature predicted by Deep DNAshape (Li *et al*. 2024). These two models showed comparable performance, with mean *R^2^* values of 0.527 and 0.538, respectively, and the difference did not reach conventional statistical significance (*P *= 0.0563; Fig. 4B). This comparison suggests that DNA breathing carries predictive information comparable in magnitude to an individual DNA shape feature such as MGW, while reflecting a different physical property: MGW describes minor-groove geometry, whereas DNA breathing captures dynamic bp opening propensity.

We then compared the breathing-based model with models incorporating 13 DNA structural features predicted by Deep DNAshape. These features included seven intra-bp and six inter-bp shape parameters. The model using 1-mer sequence features together with these 13 structural features showed substantially higher predictive performance than the model using 1-mer sequence features together with breathing features, with mean *R^2^* values of 0.661 and 0.527, respectively (Fig. 4C). This result is expected because the 13 structural features predicted by Deep DNAshape represent a broader set of sequence-dependent DNA shape properties than the two breathing features used here. Thus, DNA breathing should not be viewed as a replacement of DNA shape features.

Instead, the more relevant question is whether breathing provides information complementary to DNA shape. When breathing features were added to the model combining 1-mer sequence features with the 13 structural features, the combined model showed a small but statistically significant improvement: the mean *R^2^* increased from 0.661 to 0.664, and *R^2^* increased for 28 of 38 TFs (*P *= 0.0064; Fig. 4D). This result suggests that DNA breathing provides residual predictive information after accounting for sequence composition and DNA shape.

As complementary analyses, we compared DNA breathing features alone with sequence and DNA shape features and tested whether breathing adds information beyond MGW (Fig. 5). DNA breathing features alone showed substantially lower predictive performance than the 1-mer sequence model, with mean *R^2^* values of 0.132 and 0.503, respectively (*P *= 7.28 × 10^−^¹²; Fig. 5A). Breathing features alone also performed much worse than the full set of 13 structural features predicted by Deep DNAshape, which had a mean *R^2^* of 0.647 (*P *= 7.28 × 10^−^¹²; Fig. 5B). In contrast, breathing features alone showed performance measures comparable to MGW alone, with mean *R^2^* values of 0.132 and 0.131, respectively (*P *= 0.9657; Fig. 5C). Moreover, adding breathing features to the model combining 1-mer sequence features with MGW improved prediction, increasing the mean *R^2^* from 0.538 to 0.548, with improvement observed for 28 of 38 TFs (*P *= 0.0130; Fig. 5D). These results further support the view that DNA breathing is not sufficient to replace sequence or the full set of DNA shape features but provides predictive information comparable to an individual shape feature and partly distinct from MGW.

**Figure 5:**
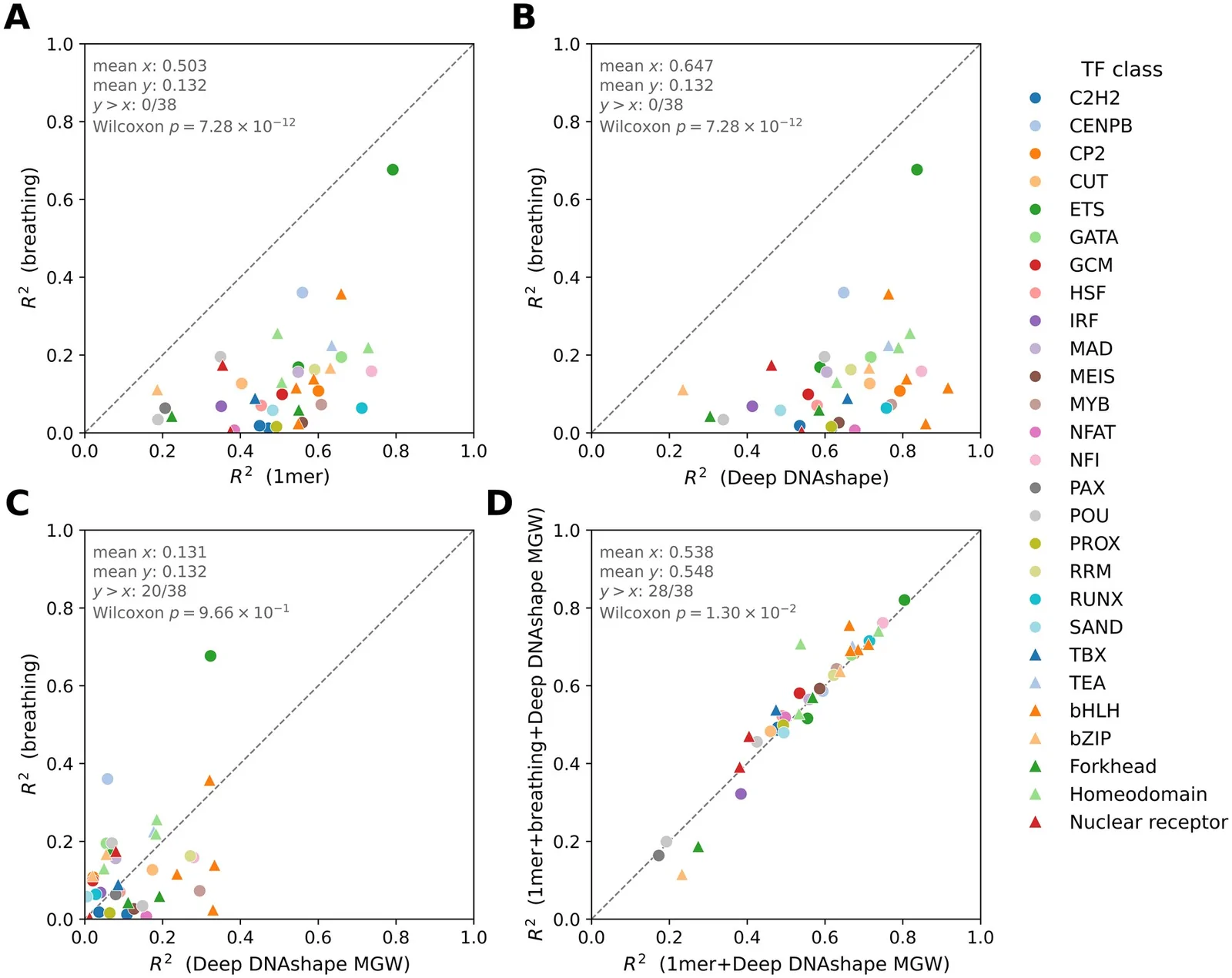
Additional model comparisons between DNA breathing, sequence, and DNA shape features. (A) Predictive performance of only breathing features compared with the 1-mer sequence model. (B) Predictive performance of only breathing features compared with the full set of DNA shape features predicted by Deep DNAshape. (C) Predictive performance of breathing features alone compared with the MGW feature predicted by Deep DNAshape. (D) Predictive performance of the 1-mer + breathing + MGW model compared with the 1-mer + MGW model. Each data point represents one TF, colored by TF class. Dashed lines indicate equal performance between the two compared models. These comparisons show that DNA breathing alone is not sufficient to replace sequence or full DNA shape features but carries predictive information comparable to MGW and provides additional information beyond MGW.

As additional model comparisons, we examined how DNA shape features contribute to TF-binding prediction both in models without DNA breathing features and in models that include DNA breathing features (Figure S7, available at supplementary material  *Bioinformatics Advances* online). In models without breathing features, adding MGW to the 1-mer model improved prediction, increasing the mean *R^2^* from 0.503 to 0.538, with improvement observed for 36 of 38 TFs (*P *= 5.03 × 10^−7^; Figure S7A, available at supplementary material  *Bioinformatics Advances* online). Adding the full set of 13 structural features predicted by Deep DNAshape to the 1-mer model produced a larger improvement, increasing the mean *R^2^* from 0.503 to 0.661, with improvement observed for 37 of 38 TFs (*P *= 1.46 × 10^−^¹¹; Figure S7B). We then asked whether DNA shape features retained predictive value when breathing features were included. Adding the 13 structural features to the 1-mer + breathing model increased the mean *R^2^* from 0.527 to 0.664, with improvement observed for 37 of 38 TFs (*P *= 7.28 × 10^−^¹¹; Figure S7C, available at supplementary material  *Bioinformatics Advances* online). These comparisons show that DNA shape features, especially the full structural feature set, retain substantial predictive information even when DNA breathing features are included in the model whose performance the full model is compared to.

Together, these analyses support the interpretation that DNA breathing is a useful and complementary physical feature for modeling TF binding. At the same time, its contribution is modest and partially overlaps with established DNA shape features, consistent with the idea that breathing reflects sequence-dependent physical properties rather than an independent feature unrelated to DNA sequence or shape.

### 3.5 DNA breathing features are associated with *in-vivo* TF binding

To gain additional insight into whether DNA breathing is associated with TF binding in a complex cellular context, we extended our analysis to a larger *in vivo* TF-binding dataset derived from ChIP-seq experiments. This dataset was collected from ChIP-seq studies of 64 TFs (Zhou and Troyanskaya 2015). Among these TFs, 44 have known binding motifs, so our analysis focused on these 44 TFs (Table S2, available at supplementary material  *Bioinformatics Advances* online). For each TF, sequences with a ChIP-seq peak label in the DeepSEA-derived annotation matrix were treated as TF-bound sequences, and we included only TFs with at least 3000 peaks. Sequences from the same 200-bp DeepSEA sequence collection without a peak label for that TF were treated as putatively unbound controls (see Methods). We then performed an analysis analogous to the *in-vitro* binding analysis using the flipping probability feature: for each sequence, we computed the average flipping probability across TF motif positions with the sequence classification for DNA binding (assigning 1 for bound sequences and 0 for unbound sequences).

We observed a wide range of correlation coefficients and statistical significance across the TFs tested. MAF bZIP TF K (MAFK) showed the strongest positive correlation, whereas zinc finger and BTB domain-containing 7A TF (ZBTB7A) exhibited the strongest negative correlation with binding status (Fig. 6A). After Bonferroni correction at α = 0.05, we found that 39 out of 44 TFs showed statistically significant correlations. For most of these TFs, the correlations are negative; in other words, higher flipping probabilities at motif positions are associated with a lower probability of TF binding (see Discussion).

**Figure 6:**
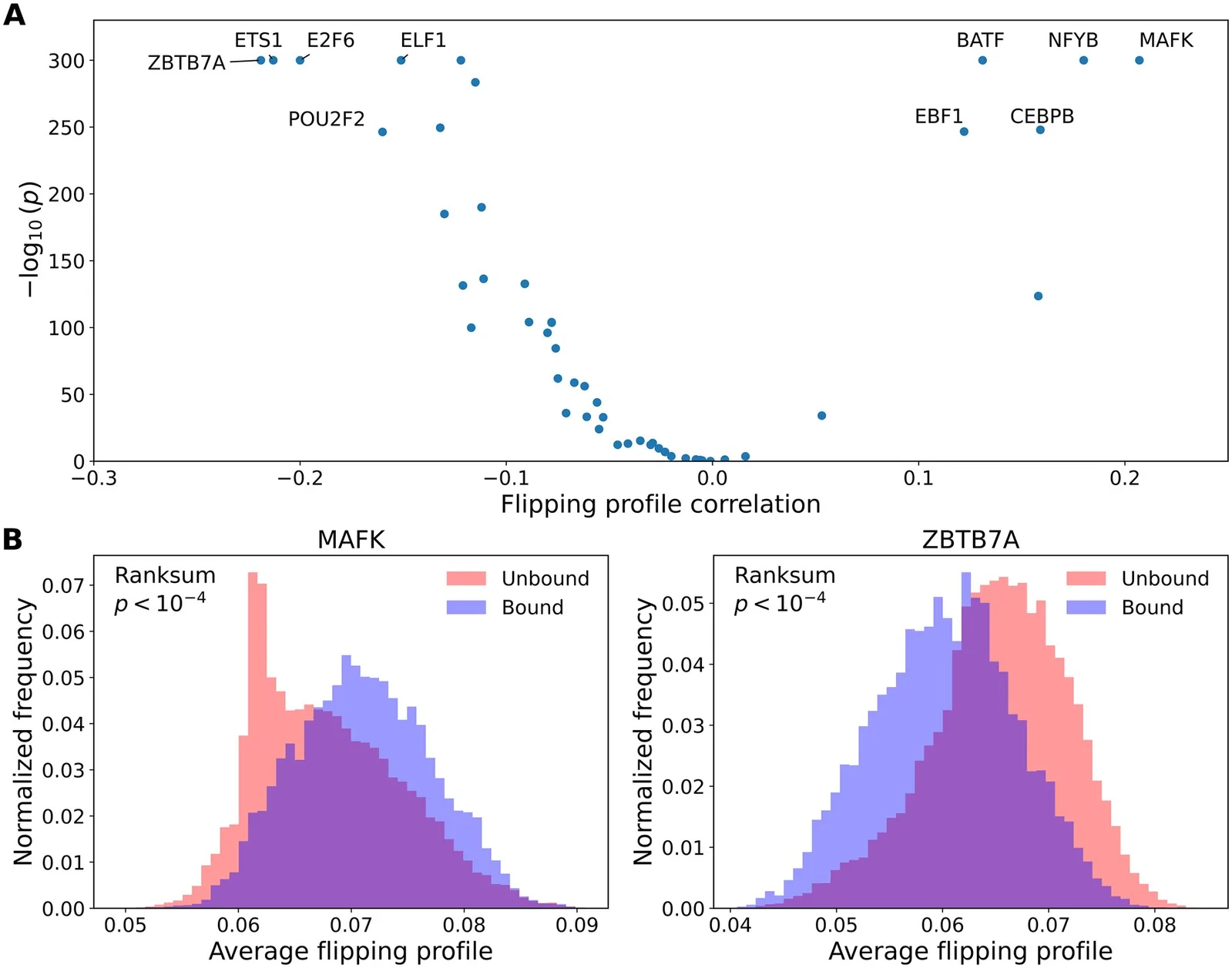
Analysis results of tests performed on a ChIP-seq dataset using 44 TFs. (A) Correlation coefficients for average flipping probability profile values at motif positions plotted against the correlation significance. (B) Histogram for the average flipping probability profile values at motifs in a sequence split according to the TF being bound (binding status = 1) or unbound (binding status = 0), with the highest positive correlation observed for MAFK (left panel) and highest negative correlation observed for ZBTB7A (right panel).

We focused here on MAFK and ZBTB7A, which showed the strongest positive and negative correlations, respectively. In MAFK-bound sequences, the average flipping probability at TF motif positions was substantially higher than in unbound sequences (Fig. 6B). Conversely, for ZBTB7A, unbound sequences showed higher average flipping probability than bound sequences. Together, these results suggest that DNA breathing features are associated with binding for many TFs, but the direction of the association varies across TFs, possibly reflecting TF-specific structural readout mechanisms.

We next explored DNA bubble probability as a second DNA breathing feature. Because computing bubble probability relies on computationally intensive molecular simulations, we limited this analysis to the 10 TFs from the flipping probability analysis above, including the five most positively and five most negatively correlated TFs. For each sequence, we selected the strongest motif match based on the motif-scanning *P*-value. We then tested if the presence of a nearby bubble, defined as a bubble within 10 bp of the motif, was correlated with TF binding status (1 for bound or 0 for unbound). For all five TFs whose flipping probability showed negative correlations with TF binding, bubble probability also showed negative correlations with TF binding (Fig. S8A, available at supplementary material  *Bioinformatics Advances* online). For the five TFs whose flipping probability showed positive correlations with TF binding, we observed varying patterns (Fig. S8A, available at supplementary material  *Bioinformatics Advances* online).

MAFK showed a strong positive association, whereas nuclear transcription factor Y subunit beta (NFYB) and CCAAT/enhancer-binding protein beta (CEBPB) showed modest negative associations. Basic leucine zipper ATF-like TF (BATF) and early B-cell factor 1 (EBF1) showed no statistically significant associations. We also repeated the analysis using alternative distance cutoffs to define nearby bubbles (5 bp and 20 bp), and the results were largely similar, indicating that the ChIP-seq bubble results were not driven by the specific motif–bubble proximity cutoff. Examining MAFK and ZBTB7A in more detail, we found that MAFK-bound sequences were more likely to contain bubbles near motifs than unbound sequences, with approximately threefold enrichment (Fig. S8A, available at supplementary material  *Bioinformatics Advances* online). In contrast, for ZBTB7A, unbound sequences were more likely to contain bubbles near motifs, suggesting that nearby bubbles may be associated with reduced binding for this TF (Fig. S8B, available at supplementary material  *Bioinformatics Advances* online). These contrasting patterns raise the possibility of TF-specific structural readout mechanisms.

Altogether, these results support the relevance of DNA breathing features in TF–DNA interactions *in vivo* and underscore the complexity of their associations with TF binding. The direction of the flipping-probability associations varied significantly across TFs, with higher flipping probabilities associated with increased binding for some TFs and reduced binding for others. The bubble-probability analysis was broadly consistent with the flipping analysis for TFs with negative association, whereas TFs with positive flipping correlations showed more bubble patterns. These differences may reflect the lower statistical power of the bubble analysis, given that DNA bubbles are relatively rare and that this analysis was limited to a subset of TFs. In addition, the precise proximity of bubbles to motif sites may influence whether they are associated with increased or reduced TF binding.

## 4 Discussion and conclusions

We presented a comprehensive analysis of the role of DNA breathing and shape in protein–DNA interactions, focusing on TF binding in both *in vitro* and *in vivo* contexts. Because TF–DNA interactions underlie much of gene regulation, this work provides an expanded perspective on how dynamic physical properties of DNA may contribute to regulatory specificity. Using both *in vitro* and *in vivo* TF-binding datasets, our analyses show that DNA breathing describes mechanical properties of DNA that are complementary to and distinct from DNA shape. DNA flipping probability at motif positions correlated with TF binding affinities, and DNA bubble probability near motifs also correlated with binding. The sign and magnitude of these associations, however, seem to vary across TFs, suggesting that DNA breathing may be linked to TF-specific modes of structural readout mechanisms.

An important point emerging from our analysis is that DNA breathing should not be interpreted as a feature independent of DNA sequence. Rather, breathing represents a physical consequence of sequence-dependent interactions, including intra-bp hydrogen bonding and nearest-neighbor stacking. This is consistent with our analyses of GC content and YpR dinucleotide steps. GC content affects local duplex stability through intra-bp hydrogen bonding, whereas YpR steps can influence stacking geometry between adjacent bp and thereby local conformational flexibility. When sequences were grouped by GC content or YpR step count, the relationship between breathing features and TF binding became clearer at the aggregate level. This suggests that DNA breathing integrates multiple sequence-composition effects into a physical property that can be relevant for TF binding. At the same time, the modest correlations observed at the individual-sequence level indicate that breathing is not the only determinant of binding; motif strength, flanking sequences, DNA shape, and TF-specific recognition mechanisms are also likely to contribute.

Our comparison with DNA shape features further refines this interpretation. MGW showed only weak correlations with flipping and bubble probabilities, indicating that DNA breathing is not simply another representation of groove geometry. In contrast, ProT showed a stronger association with both breathing features. This distinction is informative because MGW describes a groove-level structural feature, whereas ProT reflects local bp geometry and stacking interactions. The stronger relationship with ProT suggests that DNA breathing is more closely related to the local base-pairing geometry and stability. ProT indicates regions with reduced base-pairing stability, which in turn enables DNA breathing. In this sense, DNA breathing may be viewed as a dynamic extension of sequence-dependent DNA shape: conventional DNA shape features describe average structural geometry, whereas breathing features describe the propensity to sample transiently open or extrahelical conformations.

The machine-learning analysis of the HT-SELEX data further supports this intermediate interpretation. Because HT-SELEX binding assays provide binding profiles across many TFs in a controlled *in vitro* setting, they allowed us to test whether DNA breathing features improve TF-binding prediction beyond sequence and shape features. Adding DNA breathing features to a simple sequence-based (1-mer) model improved prediction performance, suggesting that breathing captures information not fully represented by sequence composition alone. However, the full DNA-shape model performed better than the breathing-only model, indicating that DNA breathing is not a complete substitute for established DNA shape descriptors. Importantly, adding breathing features to the DNA-shape model still produced a modest improvement, suggesting that breathing partially overlaps with static DNA shape features but also retains complementary dynamic information relevant to TF binding. Thus, the HT-SELEX-based machine-learning results support the view that DNA breathing is neither fully independent of sequence and shape nor redundant with them but rather provides a physically interpretable layer linking sequence composition, local DNA mechanics, and TF recognition.

Our results generally support a role for DNA breathing in TF–DNA interactions, but several limitations should be considered. We found that the results based on flipping and bubble probability features were broadly consistent. This agreement is expected because both features reflect related aspects of local duplex opening: sequence contexts that lower the energetic cost of disrupting intra-bp hydrogen bonds and nearest-neighbor stacking interactions may increase both single-bp opening and multi-bp bubble formation.

At the same time, the two features capture different aspects of DNA breathing. Flipping probability describes local bp-level opening and conformational flexibility, whereas bubble probability describes the tendency for several adjacent bp to open simultaneously, leading to transient single-stranded regions. Thus, agreement between the two features supports a shared physical basis, while differences between them may reflect the distinct spatial scales of local flipping and extended bubble formation.

Another limitation of our analysis is the possible difference between *in vitro* and *in vivo* settings. The ChIP-seq analysis used standardized 200-bp DeepSEA sequence windows, and we did not systematically test alternative peak-centered window lengths or less curated ChIP-seq sequence sets. Because breathing features were summarized locally at motif positions or near motif matches, the signal is expected to mainly reflect motif-proximal breathing, although window boundaries could still affect EPBD simulations, bubble detection, and negative-control contexts. In addition, the DeepSEA-derived negative examples were not explicitly matched to positive examples by GC content or distance to transcription start sites, although they were drawn from the same DeepSEA sequence collection rather than from random genomic background. In the ChIP-seq analysis, most TFs showed negative correlations between DNA breathing in the motifs and TF binding. One possible complication *in vivo* is the presence of nucleosomes, which can modulate access to TF motifs and thereby complicate the relationship between DNA breathing and TF binding. For example, sequence contexts with higher breathing propensity may also have mechanical properties that influence nucleosome occupancy or positioning near TF motifs. If such contexts promote nucleosome occupancy, they could reduce TF access to DNA and produce an apparent negative association between DNA breathing and TF binding. This may help explain the overall negative correlations observed for many TFs. On the other hand, TFs that act as “pioneer factors” may be less susceptible to this effect because they can bind nucleosomal DNA or promote nucleosome displacement. Consistent with this possibility, several TFs with positive correlations have been reported to have pioneer-like activity, including NFYB as part of the NF-Y complex and EBF1 (Boller *et al*. 2016, Dolfini *et al*. 2016).

It is worth pointing out several directions for future studies. First, it would be interesting to develop a quantitative model of TF–DNA interactions that explicitly incorporates DNA breathing. A recent study explored this possibility using deep neural networks that combine both DNA sequences and DNA breathing features (Kabir *et al*. 2024). However, this study has several limitations and flaws. The performance gain reported in that study seemed to depend on model architecture. Their best-performing sequence-only model achieved performance comparable to a model incorporating both sequence and DNA breathing features, raising doubt if DNA breathing contributes to TF binding. The study thus merited reanalysis as presented in this work. We caution that DNA breathing itself depends on the underlying DNA sequences, and the model in the previous study did not consider the dependence of DNA breathing on nucleotide sequence. Furthermore, models were not assessed and compared correctly (e.g. models combining nucleotide sequence and breathing features were compared with DNA shape features not capturing, for instance, hydrogen bonding opportunities). Specifically, the authors seemed to draw conclusions based on the comparison of linear with non-linear models, which raises concerns about the validity of their conclusions (Kabir *et al*. 2024).

Also, deep learning models are notoriously difficult to interpret. It would be interesting to apply strategies for interpretability in protein–DNA binding (Mitra *et al*. 2024) or develop models based on biophysical principles, for example, a model where the free energy of TF–DNA binding at any position of the motif depends on DNA breathing features at that position. It would further be interesting to explore DNA breathing from the perspective of TF families and structure. For example, using the structure of a DNA binding interface with a TF (Sagendorf *et al*. 2024) may allow one to assess to what extent binding may be influenced by DNA bubbles. These complementary approaches may help explain our finding that the role of DNA breathing seems to vary across TFs.

Another promising direction is to extend this type of analysis beyond TFs to other DNA-binding proteins and enzymes. DNA breathing may be particularly relevant for proteins whose recognition or catalytic mechanisms involve local bp opening, strand separation, or base flipping. Base flipping is a well-established mechanism in DNA-modifying and DNA-repair enzymes, including DNA methyltransferases and DNA glycosylases, where individual bases are displaced from the helix to allow direct chemical access by the protein (Klimasauskas *et al*. 1994, Slupphaug *et al*. 1996, Roberts and Cheng 1998). CRISPR-associated nucleases may also provide useful targets for this type of analysis. A recent structural study showed that Cas12a bends DNA and induces transient nucleotide flipping near the PAM-proximal region during target interrogation, exposing nucleobases for RNA–DNA hybridization (Soczek *et al*. 2025). Consistently, biochemical and SELEX-seq analyses showed that PAM-adjacent DNA flexibility and bubble formation can tune Cas12a off-target binding (Allen *et al*. 2025). These systems provide attractive test cases because local DNA opening is directly connected to protein binding, target interrogation, or catalysis. Applying DNA breathing-based models to enzymes and CRISPR systems may therefore help determine whether intrinsic breathing propensity predicts binding specificity, catalytic efficiency, or off-target recognition.

## Supplementary Material

vbag287_Supplementary_Data

## Data Availability

The data for this study are available in the Open Science Framework repository at https://doi.org/10.17605/OSF.IO/UAHBJ. The code notebooks used to generate results are available in a GitHub repository at https://github.com/TsuPeiChiu/DNAbreathing.git.
