# Supplementary material for: DNA breathing and shape encode distinct mechanisms of transcription factor binding
Source: Bioinform Adv. 2026 Sep 25;6(1):vbag287. doi: 10.1093/bioadv/vbag287 (PMC13665466; doi:10.1093/bioadv/vbag287)
Supplement: Supplementary file 1 [file vbag287_supplementary_data.zip › Chiu_etal_Supplementary_Materials.pdf]

SUPPLEMENTARY MATERIALS

DNA breathing and shape encode distinct mechanisms of transcription factor binding

Tsu-Pei Chiu[1], Wei Yu Tang[1], Waqaas Butt[2], Boqiao Lai[3], Sheng Qian[2], Boian S. Alexandrov[4], Jubao Duan[5,6], Xin He[2,*], and Remo Rohs[1,7,8,9,10,11,*]

[1]Department of Quantitative and Computational Biology, University of Southern California, Los Angeles, CA 90089, United States

[2]Department of Human Genetics, University at Chicago, Chicago, IL 60637, United States

[3]Toyota Technology Institute of Chicago, Chicago, IL 60637, United States

[4]SLIC.AI LLC, Santa Fe, NM 87507, United States

[5]Center for Psychiatric Genetics, Endeavor Health Research Institute, Evanston, IL 60201, United States

[6]Department of Psychiatry and Behavioral Neuroscience, University of Chicago, Chicago, IL 60637, United States

[7]Department of Chemistry, University of Southern California, Los Angeles, CA 90089, United States

[8]Department of Physics & Astronomy, University of Southern California, Los Angeles, CA 90089, United States

[9]Thomas Lord Department of Computer Science, University of Southern California, Los Angeles, CA 90089, United States

[10]Division of Medical Oncology, Department of Medicine, University of Southern California, Los Angeles, CA 90033, United States

[11]Alfred E. Mann Department of Biomedical Engineering, University of Southern California, Los Angeles, CA 90089, United States

*Corresponding authors. Email: xinhe@uchicago.edu (X.H.); rohs@usc.edu (R.R.)

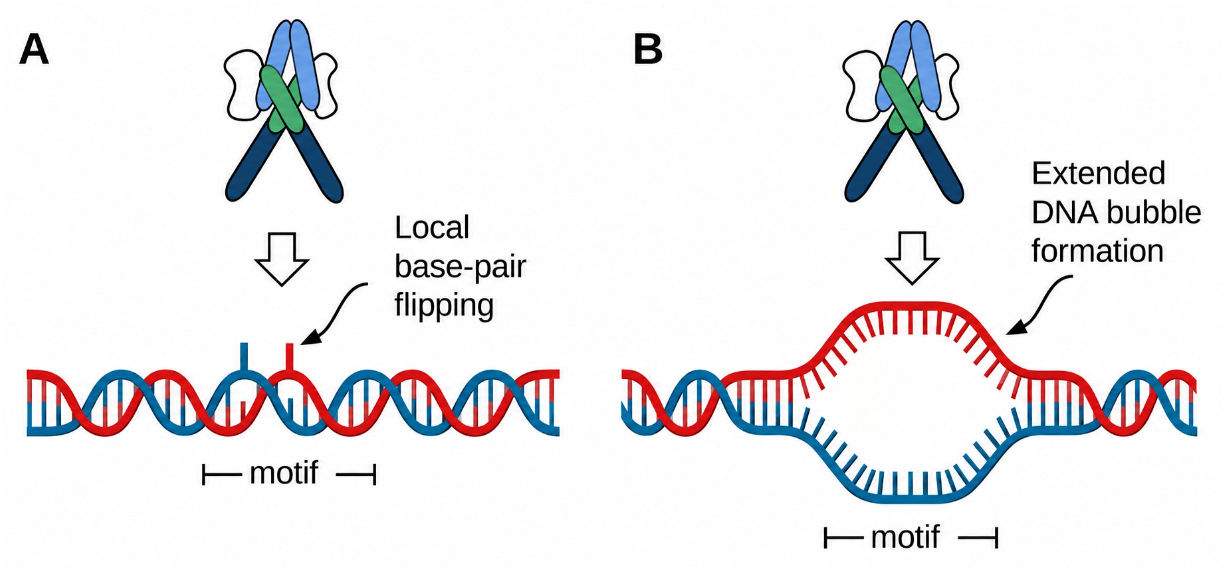

Figure S1: Schematic representation of DNA breathing features and their potential effects on TF–DNA binding. (A) Local bp flipping, in which individual bp within or near a TF motif transiently open. Such local opening may expose base-pairing edges of nucleotides and alter TF–DNA contacts. (B) Extended DNA bubble formation, in which multiple adjacent bp transiently open to form a local bubble within or near the motif. In both panels, the bracket indicates the TF motif region.

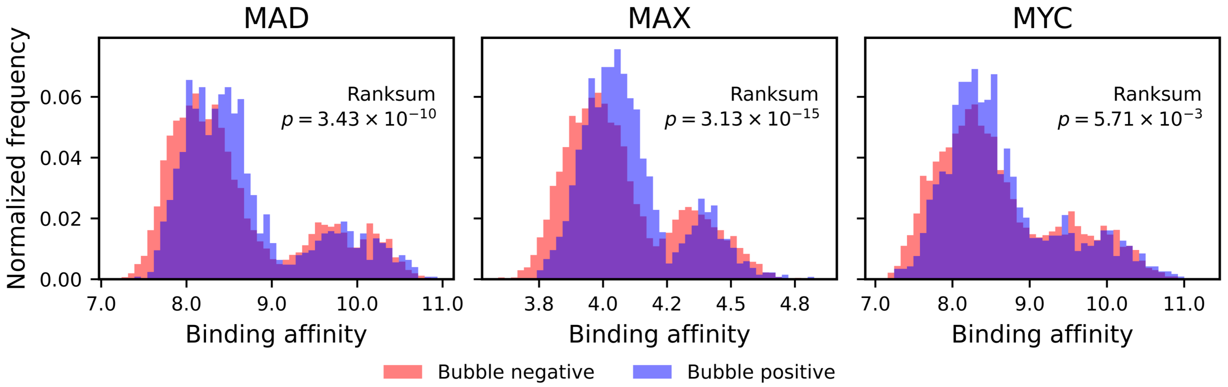

Figure S2: Binding affinity correlations with bubble presence. Histogram of the binding affinity of TF-bound sequences in the bHLH gcPBM datasets (MAD, MAX, and MYC) split according to bubble presence or absence, with normalized frequencies. Wilcoxon rank sum test performed to assess statistical significance of the difference in distributions of the two groups.

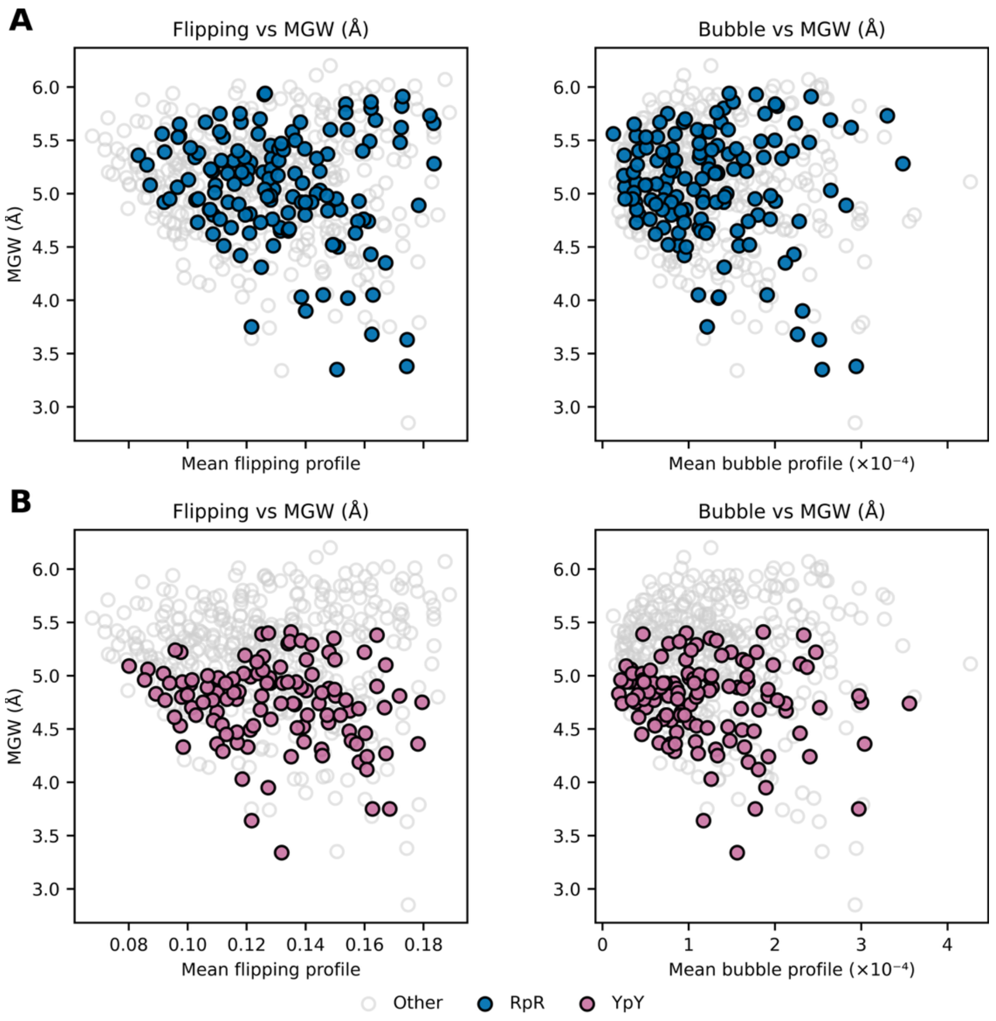

Figure S3: Distribution of RpR- and YpY-containing pentamer contexts in MGW–DNA breathing space. (A) Mean flipping probability (left panel) and mean bubble propensity (right panel) plotted against MGW, with RpR-containing pentamer contexts highlighted. (B) The same analyses with YpY-containing contexts highlighted. All other pentamer contexts are shown in gray. RpR denotes purine–purine steps and YpY denotes pyrimidine–pyrimidine steps. These control analyses show that RpR- and YpY-containing contexts are broadly distributed across the MGW–breathing space.

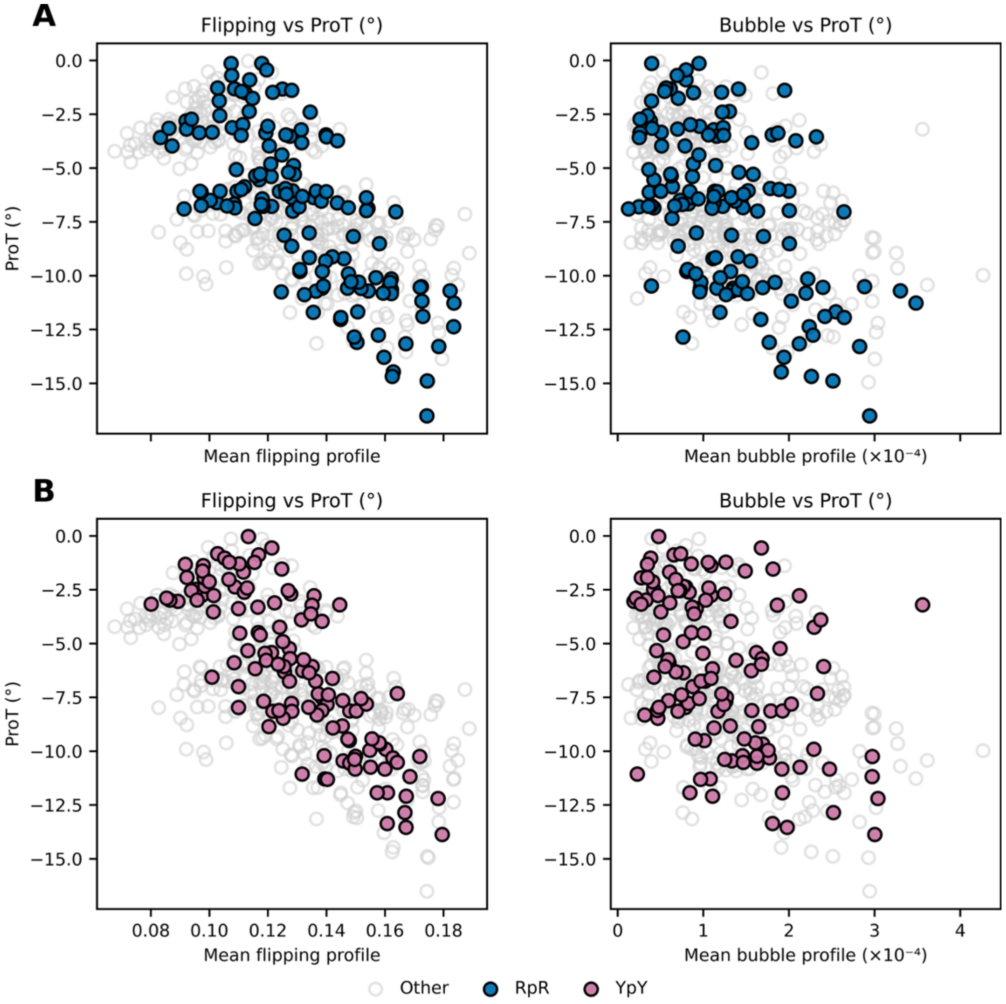

Figure S4: Distribution of RpR- and YpY-containing pentamer contexts in propeller twist (ProT)–DNA breathing space. Mean flipping probability (left panel) and mean bubble propensity (right panel) are plotted against ProT. (A) RpR-containing contexts are highlighted. (B) YpY-containing contexts are highlighted. All other pentamer contexts are shown in gray. RpR denotes purine-purine steps and YpY denotes pyrimidine-pyrimidine steps. These analyses show that RpR- and YpY-containing contexts are broadly distributed across the ProT–breathing space.

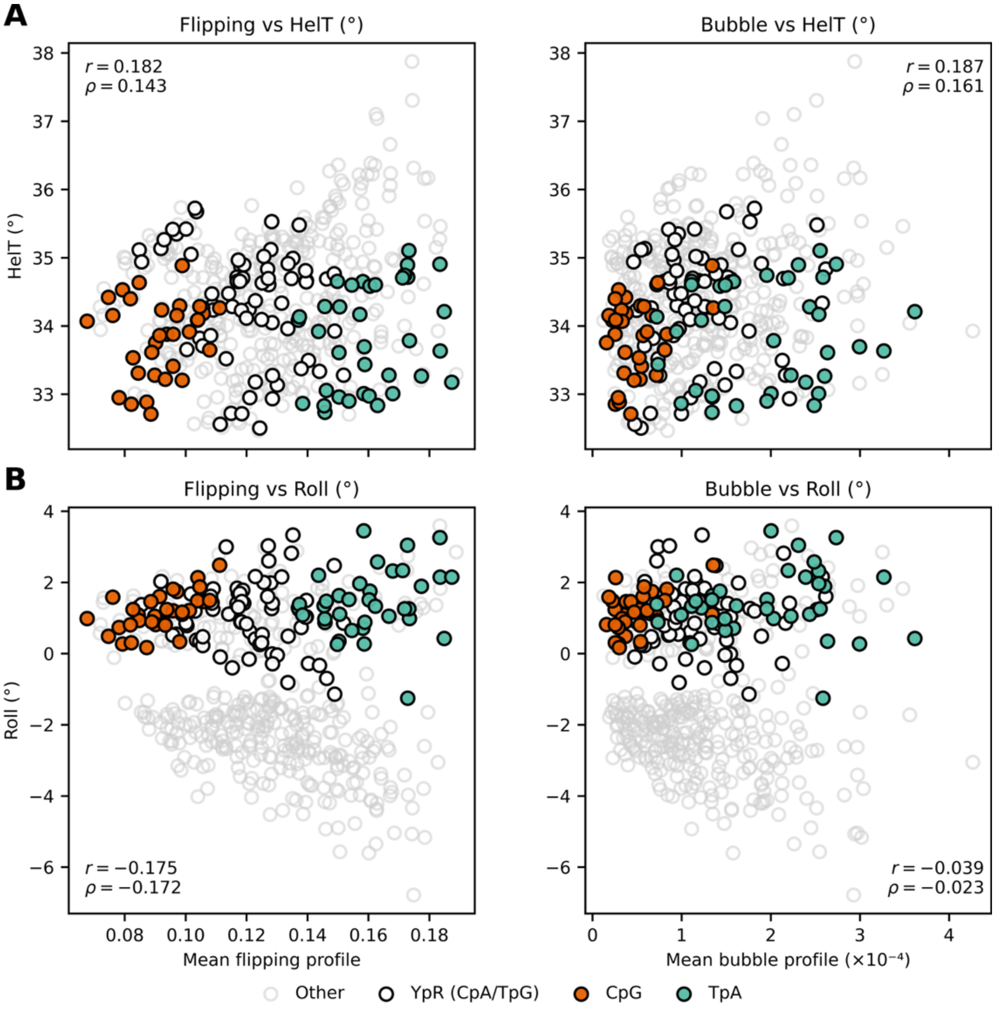

Figure S5: Relationship between DNA breathing features and base-step DNA shape parameters across pentamer sequence contexts. (A) Mean flipping probability (left panel) and mean bubble propensity (right panel) plotted against central helical twist (HelT) across 512 nonredundant pentamer contexts. (B) Mean flipping probability (left panel) and mean bubble propensity (right panel) plotted against central Roll across the same pentamer contexts. Bubble mean values are scaled by 10^{-4}. CpG- and TpA-containing contexts are highlighted in color, CpA/TpG-containing YpR contexts are shown as black open circles, and all other contexts are shown in gray. Pearson and Spearman's rank correlation coefficients are shown in each panel. HelT showed weak positive correlations with both breathing features, whereas Roll showed weak overall correlations but revealed sequence-class-specific patterns, with YpR contexts enriched in the positive Roll region.

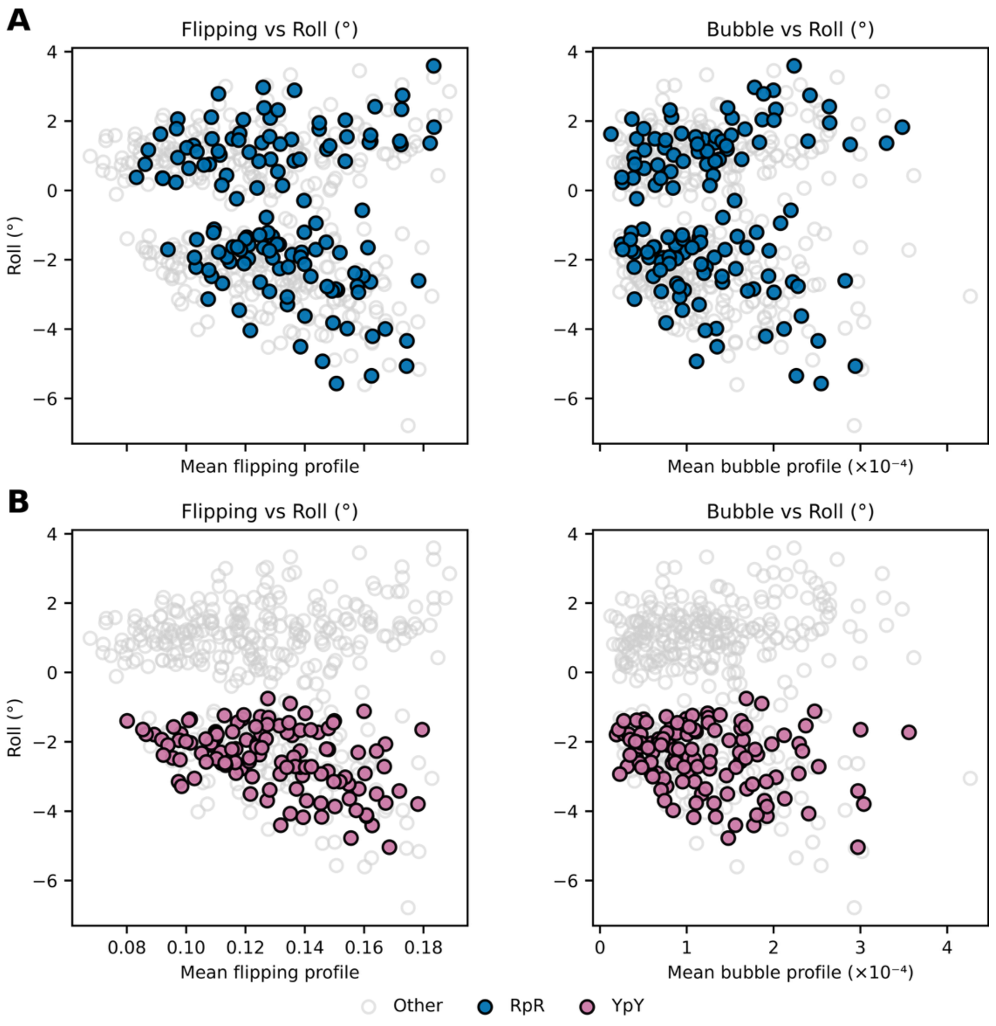

Figure S6: Distribution of RpR- and YpY-containing pentamer contexts in Roll-DNA breathing space. Mean flipping probability (left panel) and mean bubble propensity (right panel) are plotted against central Roll. (A) RpR-containing contexts are highlighted. (B) YpY-containing contexts are highlighted. Bubble mean values are scaled by 10^{-4}. All other pentamer contexts are shown in gray. RpR denotes purine-purine steps and YpY denotes pyrimidine-pyrimidine steps. These analyses show that RpR-containing contexts are broadly distributed across both positive and negative Roll regions, whereas YpY-containing contexts are enriched in the negative Roll region.

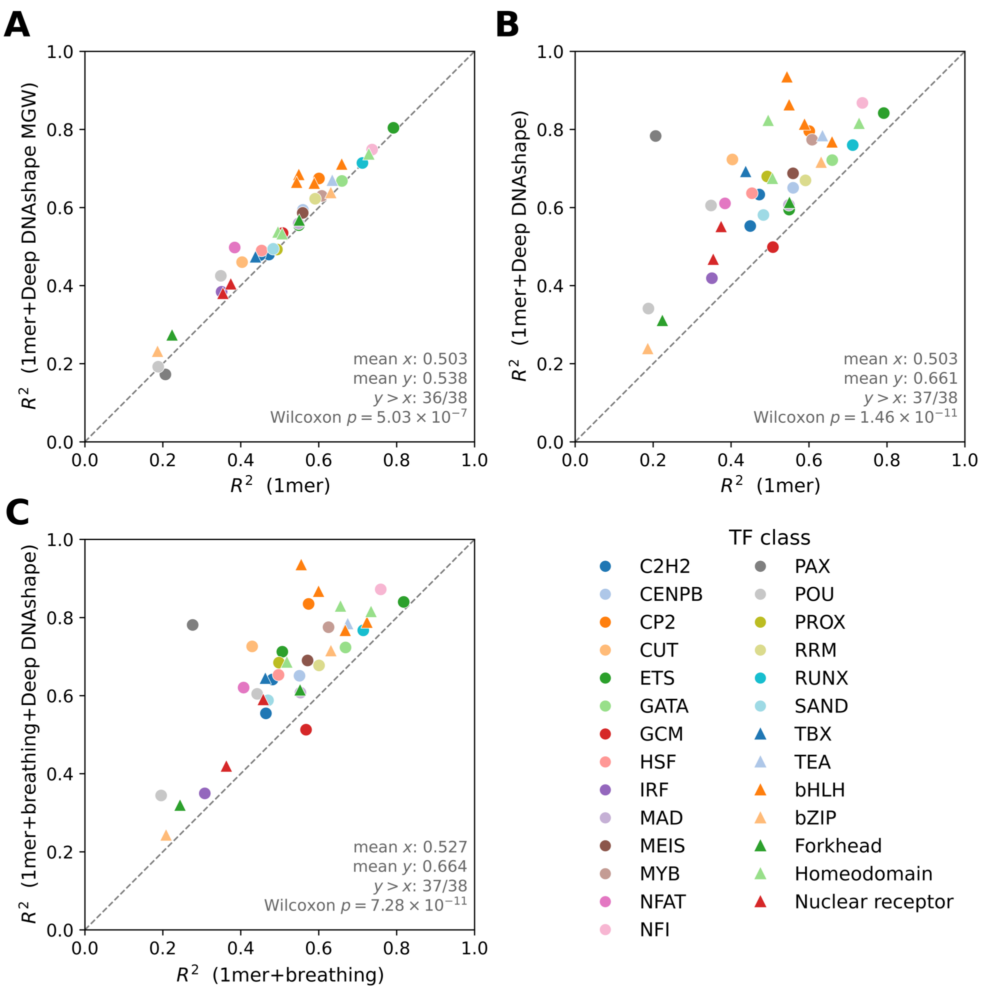

Figure S7: Additional comparisons of DNA shape features in TF-binding prediction. Comparison of 10-fold cross-validated R^2 values for 38 TFs using multiple linear regression models with different feature sets. (A) 1-mer + MGW model versus 1-mer model. (B) 1-mer + 13 DNA shape feature model versus 1-mer model. The DNA shape features were predicted by Deep DNAshape. (C) 1-mer + breathing model + 13 DNA shape feature model versus 1-mer + breathing model. The DNA shape features were predicted by Deep DNAshape. Each point represents one TF, colored and shaped by TF class. The dashed line indicates equal performance. Plot statistics report the mean R^2 along each axis, the number of TFs for which the *y*-axis model outperformed the *x*-axis model, and the *p*-value from a two-sided Wilcoxon signed-rank test.

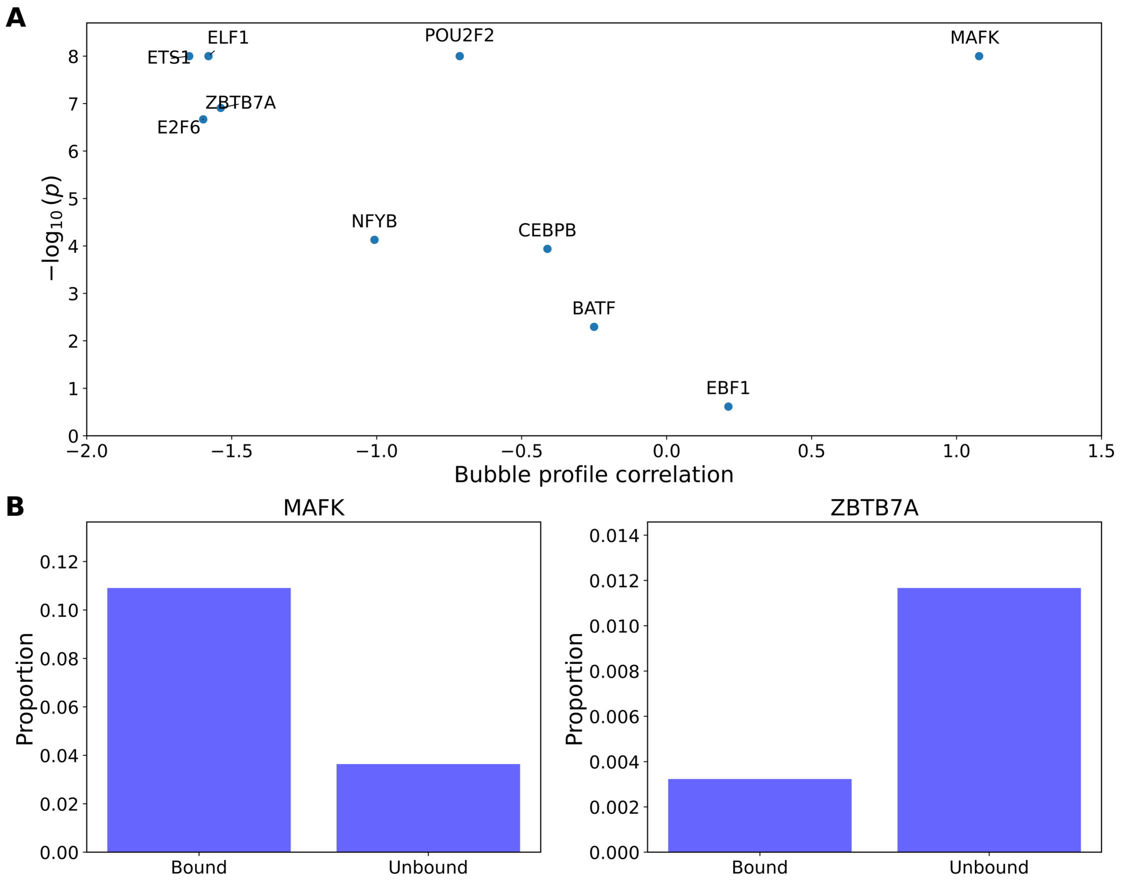

Figure S8: (A) Correlation coefficients derived through logistic regression for the distance between a motif and the nearest bubble, with distance defined as 1 if distance ≤ 10 bp, and 0 if distance > 10 bp, plotted against the correlation significance. (B) Proportion of sequences with a bubble ≤ 10 bp away from a motif in bound and unbound sequences for MAFK and ZBTB7A

Table S1: gcPBM dataset summary table. Summary table for number of sequences, number of sequences with a bubble present, and number of sequences with motif matches (total, strong, or weak) for the TFs in the gcPBM dataset. MAD does not have a motif PWM available in JASPAR and so does not have any calculated motif matches.

TF	Number of Sequences	Sequences with bubbles	Sequences with motifs	Sequences with strong motif matches	Sequences with weak motif matches
MAD	7534	1266	N/A	N/A	N/A
MAX	8568	1361	7228	1877	5351
MYC	6926	1186	5917	2636	3281

Table S2: ChIP-seq dataset summary table. Summary table for ChIP-seq data used in the analysis of flipping probability profiles. Shown are the number of ChIP-seq labels (positive or negative for bound or unbound) and number of sequences with motif matches (total, strong, weak, strong, and weak) for the TFs in the ChIP-seq dataset (Total number of sequences = 886,625). TFs without a motif PWM in JASPAR do not have any calculated motif matches.

TF	Positive Labels	Negative Labels	Sequences with motifs	Sequences with Strong Motifs	Sequences with Weak Motifs	Sequences with Strong and Weak Motifs
ATF3	1345	885280	128930	16983	115954	4007
BATF	29163	857462	129347	68926	106781	46360
BCL11A	19642	866983	NA	NA	NA	NA
BCL3	20776	865849	NA	NA	NA	NA
BCLAF1	10399	876226	NA	NA	NA	NA
BHLHE40	18507	868118	158335	20214	150287	12166
BRCA1	10018	876607	NA	NA	NA	NA
CEBPB	69979	816646	145484	22445	127335	4296
CHD2	29170	857455	NA	NA	NA	NA
CTCF	106130	780495	274424	122105	193073	40754

E2F6	26766	859859	187609	121578	95529	29498
EBF1	42988	843637	136995	93836	74472	31313
EGR1	31747	854878	130640	109636	45602	24598
ELF1	27775	858850	305486	121726	216030	32270
EP300	36078	850547	NA	NA	NA	NA
ETS1	14122	872503	294174	32504	272831	11161
FOSL1	9657	876968	130085	62819	100305	33039
FOSL2	24122	862503	178321	80303	125751	27733
FOXA1	53318	833307	185016	56338	141652	12974
FOXA2	43098	843527	286926	55083	253409	21566
GABPA	16170	870455	311670	120713	223833	32876
GATA2	19153	867472	230736	38182	203951	11397
GATA3	46530	840095	NA	NA	NA	NA
HNF4A	19826	866799	312704	41905	286306	15507
IRF4	22859	863766	237899	32723	218770	13594
JUND	21221	865404	251729	41306	234716	24293
MAFK	43832	842793	214305	83746	166218	35659
MAX	58358	828267	205717	25885	195985	16153
MEF2A	20471	866154	202955	68191	155651	20887
NFYB	19280	867345	183471	48870	147501	12900
NR2F2	25490	861135	189030	51507	146895	9372
PAX5	24817	861808	282264	35148	262289	15173
PBX3	8402	878223	242357	72765	188985	19393
PML	29972	856653	NA	NA	NA	NA
POLR2A	65533	821092	NA	NA	NA	NA
POU2F2	27074	859551	250236	41195	226594	17553

PRDM1	5367	881258	275886	87427	219287	30828
RAD21	49801	836824	NA	NA	NA	NA
REST	14275	872350	242699	76391	189363	23055
RFX5	27110	859515	162149	19442	151098	8391
RUNX3	84913	801712	191717	51292	149682	9257
SIN3AK20	26334	860291	NA	NA	NA	NA
SIX5	7676	878949	NA	NA	NA	NA
SMC3	38443	848182	NA	NA	NA	NA
SP1	20871	865754	179904	138463	69518	28077
SPI1	45251	841374	NA	NA	NA	NA
SRF	7212	879413	153825	18068	146552	10795
STAT3	20502	866123	215541	70727	169062	24248
STAT5A	13875	872750	NA	NA	NA	NA
TAF1	22303	864322	NA	NA	NA	NA
TAF7	4710	881915	NA	NA	NA	NA
TBP	28152	858473	NA	NA	NA	NA
TCF12	20167	866458	278716	91546	224068	36898
TCF7L2	6791	879834	224317	54233	183186	13102
TEAD4	43252	843373	205654	63596	154307	12249
TFAP2C	48159	838466	179160	120627	92522	33989
THAP1	3808	882817	176816	113132	83556	19872
TRIM28	21017	865608	NA	NA	NA	NA
USF1	23206	863419	162794	53551	133893	24650
USF2	13887	872738	170456	26251	151203	6998
YY1	37554	849071	NA	NA	NA	NA
ZBTB33	2209	884416	118730	17535	114756	13561

ZBTB7A	26635	859990	239817	69919	193164	23266
ZNF143	7697	878928	186410	47465	147538	8593

Table S3: Bubble propensity analysis ChIP-seq dataset subset summary table. Summary table of the ChIP-seq data used in the bubble propensity analysis. Shown are the number of ChIP-seq labels (positive or negative for bound or unbound), number of sequences with a bubble present, and number of sequences with motif matches (total, strong, weak, strong, and weak) for the TFs in the subset of the total ChIP-seq dataset that we rendered bubble propensity features for (Total number of sequences = 35,331).

TF	Positive Labels	Negative Labels	Sequences with Bubbles	Sequences with motifs	Sequences with Strong Motifs	Sequences with Weak Motifs	Sequences with Strong and Weak Motifs
MAFK	3596	31735	13938	9708	4217	7243	1752
NFYB	3166	32165	13938	8128	2582	6300	754
CEBPB	3130	32201	13938	4716	555	4260	99
BATF	5130	30201	13938	6114	3874	4857	2617
EBF1	7641	27690	13938	7016	5005	3767	1756
ZBTB7A	4620	30711	13938	14328	5752	11238	2662
ETS1	5733	29598	13938	14873	2898	13257	1282
E2F6	6392	28939	13938	11501	8053	6036	2588
POU2F2	8135	27196	13938	7227	1409	6305	487
ELF1	8974	26357	13938	14291	6306	9892	1907

Table S4: Exact JASPAR2022 motif profiles used for FIMO scanning. Table S4 is provided as a separate Excel file. The workbook lists the exact versioned JASPAR matrix identifiers used in the gcPBM and ChIP-seq analyses. Separate sheets provide the motifs used for each dataset.
